# Erythropoietin receptor regulates tumor mitochondrial biogenesis through iNOS and pAKT

**DOI:** 10.3389/fonc.2022.976961

**Published:** 2022-08-16

**Authors:** Mostafa A. Aboouf, Franco Guscetti, Nadine von Büren, Julia Armbruster, Hyrije Ademi, Maja Ruetten, Florinda Meléndez-Rodríguez, Thomas Rülicke, Alexander Seymer, Robert A. Jacobs, Edith M. Schneider Gasser, Julian Aragones, Drorit Neumann, Max Gassmann, Markus Thiersch

**Affiliations:** ^1^ Institute of Veterinary Physiology, Vetsuisse Faculty, University of Zurich, Zurich, Switzerland; ^2^ Zurich Center for Integrative Human Physiology (ZIHP), University of Zurich, Zurich, Switzerland; ^3^ Center for Clinical Studies, Vetsuisse Faculty, University of Zurich, Zurich, Switzerland; ^4^ Department of Biochemistry, Faculty of Pharmacy, Ain Shams University, Cairo, Egypt; ^5^ Institute of Veterinary Pathology, Vetsuisse Faculty, University of Zurich, Zurich, Switzerland; ^6^ PathoVet AG, Pathology Diagnostic Laboratory, Tagelswangen, Switzerland; ^7^ Hospital Universitario Santa Cristina, Autonomous University of Madrid, Madrid, Spain; ^8^ Department of Biomedical Sciences, University of Veterinary Medicine Vienna, Vienna, Austria; ^9^ Department for Sociology and Social Geography, Paris Lodron University of Salzburg (PLUS), Salzburg, Austria; ^10^ Department of Human Physiology & Nutrition, University of Colorado Colorado Springs (UCCS), Colorado Springs, CO, United States; ^11^ Center of Neuroscience Zurich (ZNZ), University of Zurich, Zurich, Switzerland; ^12^ Department of Cell and Developmental Biology, Sackler Faculty of Medicine, Tel Aviv University, Tel-Aviv, Israel

**Keywords:** erythropoietin receptor, tumor metabolism, mitochondrial biogenesis, nitric oxide (NO), respirometry, OXPHOS, VDAC1

## Abstract

Erythropoietin receptor (EPOR) is widely expressed in healthy and malignant tissues. In certain malignancies, EPOR stimulates tumor growth. In healthy tissues, EPOR controls processes other than erythropoiesis, including mitochondrial metabolism. We hypothesized that EPOR also controls the mitochondrial metabolism in cancer cells. To test this hypothesis, we generated EPOR-knockdown cancer cells to grow tumor xenografts in mice and analyzed tumor cellular respiration *via* high-resolution respirometry. Furthermore, we analyzed cellular respiratory control, mitochondrial content, and regulators of mitochondrial biogenesis *in vivo* and *in vitro* in different cancer cell lines. Our results show that EPOR controls tumor growth and mitochondrial biogenesis in tumors by controlling the levels of both, pAKT and inducible NO synthase (iNOS). Furthermore, we observed that the expression of EPOR is associated with the expression of the mitochondrial marker VDAC1 in tissue arrays of lung cancer patients, suggesting that EPOR indeed helps to regulate mitochondrial biogenesis in tumors of cancer patients. Thus, our data imply that EPOR not only stimulates tumor growth but also regulates tumor metabolism and is a target for direct intervention against progression.

## Introduction

EPOR is expressed in non-hematopoietic tissues including cancer cells (1), suggesting that it plays a role beyond erythropoiesis in malignant tissues. Indeed, the expression of EPOR has raised concerns about the safety of EPO treatment in cancer patients with anemia because EPO may stimulate cancer cell survival and tumor progression. In lung cancer patients, coexpression of EPO and EPOR is associated with poor survival (2). Several other clinical studies have reported reduced survival rates in EPO-treated cancer patients (3). In preclinical studies, EPO has been shown to induce the proliferation of different cancer cells, such as colorectal (4) or breast cancer (4–7), and may stimulate the conversion of non-stem breast cancer cells into breast cancer-initiating cells (7, 8). In contrast, some cancer cell lines have been reported to be non-responsive to EPO, although they express EPOR transcripts, but not functional EPOR (9, 10). The notion that some tumors express functional EPOR is based on the binding of EPO to EPOR in cancer cells *in vitro* and *in vivo* (11). Moreover, the loss of EPOR delays *in vitro* breast cancer cell growth (9) as well as *in vivo* tumor growth in breast cancer (5) and glioma models (12). These data demonstrate the importance of determining EPOR function in tumors, and not merely its expression level.

For example, in fat tissue, the loss of EPOR results in obesity and other metabolic syndrome phenotypes, suggesting that EPOR helps regulate energy homeostasis (13, 14). Furthermore, EPO not only increases red blood cell mass in healthy young men but also improves the respiratory potential in skeletal muscles (15), increasing their ability to use oxygen to drive ATP production. Cardiomyocytes show increased mitochondrial biogenesis after EPO treatment due to the induced expression of endothelial nitric oxide synthase (eNOS) (16), which most likely results in the overproduction of nitric oxide (NO). The contribution of NO to mitochondrial biogenesis is exemplified by a reduced number of mitochondria in eNOS-deficient mice (17). In addition to eNOS, neuronal nitric oxide synthase (nNOS) and inducible nitric oxide synthase (iNOS) produce NO to regulate mitochondrial biogenesis (17, 18). NO is also a physiological regulator of cellular respiration that interacts with the five complexes of the mitochondrial oxidative phosphorylation system (OXPHOS) (19, 20). The OXPHOS inhibition by NO could directly support malignant cells by promoting increased reliance on glycolytic metabolism (21). High expression levels of all three NOS isoforms in human tumors and presumably elevated NO levels correlate with malignancy and poor survival in human patients (22). Whereas the expression of eNOS and nNOS mainly depends on calcium levels (22), iNOS expression can be induced by cytokines in malignant cells (23).

We asked whether the activation of the EPO/EPOR axis controls mitochondrial metabolism in cancer cells by inducing NOS expression, and thereby regulating mitochondrial biogenesis. We used EPOR knockdown cancer cells to generate *in vivo* xenografts and to analyze the role of EPOR in the control of cellular respiration in tumors with high-resolution respirometry and mitochondrial biogenesis by using *in vitro* and *in vivo* cell biological methods.

## Material and methods

### Cancer cell line and cell culture

Human A549 non-small lung cancer cells (ATCC) were cultured in Minimum Essential Medium (MEM) (ThermoFisher Scientific), murine Lewis lung carcinoma cell line LLC1 (ATCC) were cultured in RPMI 1640 medium (ThermoFisher Scientific), and human MCF-7, epithelial adenocarcinoma-derived breast cancer cells (ATCC) were cultured in Eagle’s Minimum Essential Medium (EMEM) (ATCC). Cells were transfected using Polyjet (SignaGen#SL100688, MD, USA) and vectors for huEPOR and AKT1. For EPOR expression we used a custom-made plasmid expressing mCherry and EPOR (NM_000121.4) (VectorBuilder, Hong Kong, China) and for AKT1 expression we used a 901 pLNCX myr HA Akt1 plasmid, which was a gift from William Sellers (Addgene plasmid # 9005; http://n2t.net/addgene:9005) (24). Cells were analyzed 72 h after transfection. To inhibit pAKT and NOS, we used 5 µM of API-1 (Sigma Aldrich #SML1342, Switzerland) and 200 µM of L-NAME (abcam #ab120136). Cells were treated with either or both reagents for 72h followed by harvesting for downstream analysis. Mitochondria in live cells were stained with 20 nM Mitotracker Green FM (Invitrogen #M7514, Switzerland). Pictures were captured using the EVOS FL Auto imaging system and analyzed by ImageJ.

### Generation of stable EPOR knockdown and iNOS re-expressing cells

Human EPOR (sc‐37092‐V) and control (sc‐108080) shRNA lentiviral particles (Santa Cruz Biotechnology, Dallas, TX, USA) were used to generate stable A549‐shEPOR knockdown cells and their corresponding A549‐shSCR control cells. Infected cells were selected with 1 µg/ml puromycin. We single-seeded A549-shSCR and A549-shEPOR cells to obtain individual clones. shEPOR clones were further incubated with custom-made lentiviral particles (VectorBuilder, Hong Kong, China) to stably express either mCherry or iNOS with a neomycin resistance marker. Infected cells were selected by the neomycin analog 900 µg/ml G418, Geneticin (ThermoFischer, Switzerland) for 10 days, and iNOS expression was confirmed by qPCR.

### Animal handling and study design

Mouse experiments were performed in accordance with the Swiss animal law and with the approval of the ethical committee of the respective local authorities (Kanton Zurich). Hsd : Athymic Nude-Foxn1^nu^ (8-9 weeks old) male mice (Envigo, Netherlands) were kept in a pathogen-free mouse barrier facility (22 ± 5°C in a 12 h light/dark cycle; standard rodent chow (Kliba Nafag, #3436) and water ad libitum). We injected 3x10^6^ cells in a 1:6 Matrigel-PBS solution into the rear right flank. Five different cell lines, namely wild-type, shSCR1, shSCR2, shEPOR1, and shEPOR2 cells, were injected into groups of 16 nude mice. Seven days after tumor cell injection, mice were cage-wise split into two groups of eight animals. Subsequently, eight mice were intraperitoneally injected with 300 U/kg EPO (Epoetin-beta; Recormon^®^, Roche) (6, 25) and the other eight mice were injected with saline throughout the entire experiment. Additional confounders were not identified and controlled for. Tumor size was calculated V=1/2*Length*(Width)^2^ after measuring the length (largest tumor diameter) and width (perpendicular tumor diameter) with a caliper (26). Mice were euthanized with CO_2_ and blood for hemoglobin measurements and hematocrit (ABL 800 Flex, Radiometer) was retrieved from the right heart ventricle.

### High-resolution respirometry

Protocols for cellular respiration of tumor tissue were adopted from previous studies (27, 28) and are described in detail in the **Supplemental File**. All chemicals were obtained from Sigma-Aldrich (Switzerland). Briefly, fresh tumor biopsy mass was collected (wet weight, mg) and tissue respiration was measured in mitochondrial respiration buffer Miro06 (Miro05 + 280 iU/ml catalase) (27) at 37°C using the high-resolution Oxygraph-2k (Oroboros, Innsbruck, Austria). To measure mass-specific respiration all parameters were normalized to the wet weight of the tissue biopsies. L_N_: Leak respiration was measured after the addition of 2 mM malate and 0.2 mM octanoyl carnitine. P_ETF_: Fatty acid oxidative capacity through electron-transferring flavoprotein (ETF) was measured after adding 5 mM ADP. P_C1_: Submaximal state 3 respiratory capacity specific to complex I was induced by adding 5 mM pyruvate and 10 mM glutamate. P: Maximal state 3 respiration, oxidative phosphorylation capacity was measured after the addition of 10 mM succinate. ETS: maximal electron transport system capacity was measured by decoupling ATP synthase by repetitively adding 0.5 µM Carbonyl cyanide 4-(trifluoromethoxy)-phenylhydrazone until maximal oxygen consumption rates were achieved. P_C2_: To measure the electron flow specific to complex II, we added 0.5 µM rotenone to inhibit complex I. We then added 2.5 µM antimycin A to inhibit complex III and to determine the residual, non-mitochondrial oxygen consumption, which was used for correcting the aforementioned measurements. COX: 2 mM ascorbate and 0.5 mM TMPD were simultaneously added to assess cytochrome c oxidase (COX) complex IV activity, which correlates with mitochondrial volume density (29) and was used to transform mass-specific respiration into mitochondria-specific respiration.

### Western blotting

Protein lysates of cells and tissues were separated by SDS-PAGE and then blotted onto a nitrocellulose membrane (GE Healthcare, #10600002). Membranes were blocked with 5% milk (Rapidlait, Migros Switzerland), followed by incubation with primary antibodies (**Supplemental Table 1**) at 4°C overnight. Membranes were then incubated with HRP conjugated secondary antibodies (**Supplemental Table 1**). Bands were visualized using Super Signal West Femto Maximum Sensitivity Substrate (ThermoFisher Scientific) and recorded with FUJIFILM Intelligent Darkbox Las-3000.

### RNA extraction and mRNA expression analyses

10-20 mg of tissue were used to extract RNA using the ReliaPrep RNA Tissue Miniprep System (Promega, #Z6110). First-strand cDNA was synthesized using RevertAid First Strand cDNA Synthesis Kit (ThermoFisher Scientific, #K1622). Samples (5 ng/µl cDNA) were analyzed with a semi-quantitative real-time PCR (qRT-PCR) (7500 Fast Real-Time PCR System, ThermoFisher Scientific) using SYBR Green (ThermoFisher Scientific, #A25741). Primers for mRNA expression analyses were designed by primer 3 (30) to amplify either human or murine genes without cross-specificity (**Supplemental Table 2**). mRNA expression levels were calculated using the DDCt method (31, 32).

### DNA extraction and mitochondrial copy number

The amount of mitochondrial DNA (mtDNA) in tissue and cells was estimated in DNA extracts by the ratio of the mitochondrial *MT-ND1* gene copy number and the nuclear *N-B2M* gene copy number (33, 34). Primers against both genes (**Supplemental Table 2**) and SYBR Green (ThermoFisher Scientific, #A25741) were used for a semi-quantitative analysis by quantitative real-time PCR (7500 Fast Real-Time PCR System, ThermoFisher Scientific). The ratio of genomic (N-B2M gene) and mitochondrial (MT-ND1) DNA was determined by the DDCt method.

### Nitrate measurements

To estimate NO levels, we measured nitrate (NO_3_^−^) and nitrite (NO_2_^-^), which are stable oxidation products and reliable markers of NO (35) in plasma of mice, by a gas phase triiodide-based chemiluminescence assay (36). We measured nitrite by injecting 50 µl plasma into the preheated (65°C) reaction chamber containing acidic triiodide (I3−) Brown’s reagent (1.65 g KI, 0.57 g I_2_, 15 ml ddH_2_O, and 200 ml glacial CH_3_COOH). The reaction chamber was purged with helium. Released NO was measured using the CLD-88 analyzer (ECO MEDICS, Durnten, Switzerland) and recorded using PowerChrom 280 system (eDAQ Pty; Spechbach, Germany). To measure nitrate, we used a cadmium-copper-based reduction kit Nitralyzer-II (World Precision Instruments, Sarasota, FL) to reduce nitrate to nitrite. After the reduction, nitrite was measured as described above and nitrate levels were estimated by the subtraction of nitrite levels before the reduction from those obtained after the conversion of nitrate to nitrite.

### Immunohistochemistry of tumor tissues

Lung cancer array sections (US Biomax Inc., MD, USA, LC121 and LC1921b) were subjected to antigen retrieval at 125°C for 2 min in EDTA buffer (pH 9.0) in a steamer, incubated overnight with a rabbit anti-VDAC1/Porin antibody (Abcam, #ab15895), 1:500 at 4°C, followed by Envision for 30 min and AEC 10 min (Agilent, K4003 & K3469). Then, the sections were incubated overnight with a rat anti-human EPOR monoclonal antibody (Genovac, #GM-1201), 1:50 at 4°C, followed by a rabbi-anti-rat antibody (Vector, BA-4000), 1:800 for 30 min, this reaction was visualized with DAB (Agilent, K3468). Slides were fully scanned (NanoZoomer 2.0-HT; Hamamatsu, Hamamatsu City, Japan) and images of individual cores were captured. The color deconvolution plugin was employed to separate channels that correspond to three determined RGB colors by the ImageJ tool. Separated stained signal areas were then isolated using the IHC toolbox plugin followed by quantification of the pixels area of the black/white picture and calculation of its ratio to the total measured tumor core area. Tumor sections of A549 xenografts were stained and processed similarly.

### Analysis of lung cancer datasets

We used the lung cancer explorer (https://lce.biohpc.swmed.edu/lungcancer/index.php#page-top) from the Quantitative Biomedical Research Center (UT Southwestern Medical Center) (37) to analyze different lung adenocarcinoma datasets from human patients. We performed a comparative analysis using the TCGA_LUAD_2016 study (56 healthy and 517 tumor samples) (38) and the Takeuchi_2006 study (5 healthy and 158 tumor samples) (39) comparing *VDAC1* mRNA expression in healthy lung tissue and lung adenocarcinomas. We further performed survival analyses to estimate the association between the overall survival of lung adenocarcinoma patients and *VDAC1* mRNA expression in three studies, namely TCGA_LUAD_2016 (38), Takeuchi_2006 (39), and Schabath_2016 (40). The cutoff for samples with high or low expression was the global mean of *VDAC1* expression and data sets were analyzed with a log-rank test.

### Data analysis

All cell biological analyses were performed blinded, and the sample IDs were known to the principal investigator. We used GraphPad Prism for generating graphs and R version 3.6.2 R Core Team (2020) for statistical analyzes. We used the student`s t-test for normally distributed data and the Mann-Whitney test for non-parametrically distributed data. Data distribution was estimated with Shapiro-Wilk and Kolmogorov Smirnov test. For multiple comparisons, we used either the Kruskal Wallis test with Dunn`s multiple comparison test for nonparametrically distributed data or a one-way ANOVA with the Bonferroni *post hoc* test. For repeated measurements, the aligned rank transformation ANOVA was used. A p-value of 0.05 was considered statistically significant. Lung cancer array stainings were analyzed by Pearson correlation.

## Results

### Knockdown of EPOR impairs tumor growth of A549 lung cancer xenografts in Foxn1nu mice

**Figure 1:**
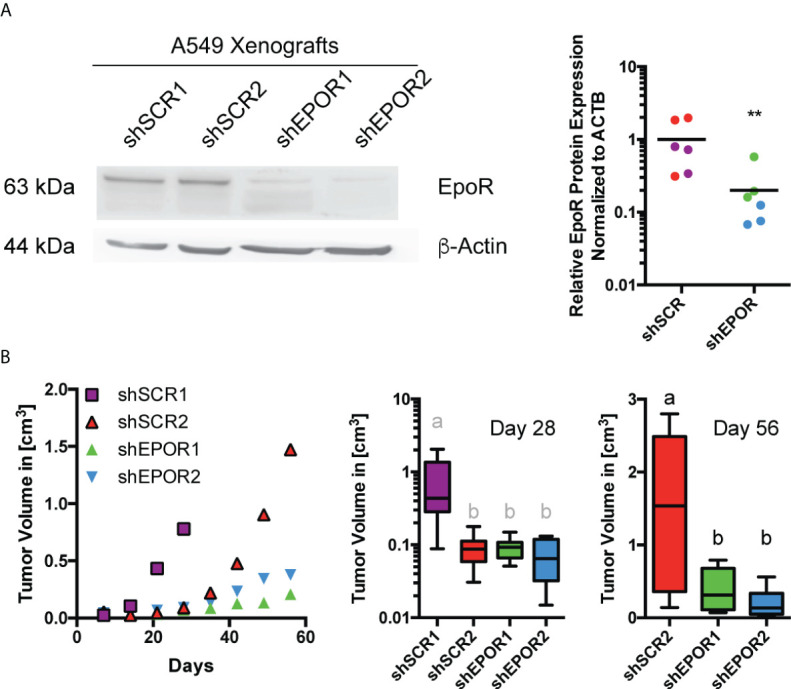
Knockdown of EPOR impairs tumor growth of A549 lung cancer xenografts in Foxn1nu mice. A549 control cells (shSCR1, purple and shSCR2, red) or A549 EPOR knockdown cells (shEPOR1, green and shEPOR2, blue) were subcutaneously injected (3 x 10^6^ cells in 100 µl PBS/Matrigel) into Foxn1nu mice. Panel **(A)** shows a representative western blot image of EPOR (63 kDA) and β-actin (44 kDA) protein expression in shSCR and shEPOR A549 tumors (left panel). Western blotting images were analyzed by MCID Analysis 7.0 and shown is relative EPOR protein expression of shSCR (purple shSCR1 tumors, red shSCR2 tumors) and shEPOR (green shEPOR1 tumors, blue shEPOR2) tumors normalized to β-actin (n=6) (right panel). Panel **(B)** shows the tumor growth curves (left panel), tumor size 28 days after tumor cell implantation (middle panel), and tumor size 56 days after tumor cell implantation (right panel) for shSCR A549 and shEPOR tumors (n=8). Please note: The middle panel has a logarithmic scale, and in the right panel, no data for shSCR1 tumors are shown because the experiment was already terminated 28 days after tumor cell implantation. Data are presented either as scattered blots with mean, as mean, or as a box plot with min to max whiskers. A Student’s t-test (black symbols), a Kruskal Wallis test with Dunn’s multiple comparison test (grey letters), or a one-way ANOVA with Bonferroni *post hoc* test (black letters) was performed (**p<0.01); letters a and b indicate groups that statistically (p<0.05) differ from each other.

The expression of EPOR in tumors of lung cancer patients is associated with poor survival (2). Therefore, we used human A549 lung cancer cells, which express EPOR (1, 11, 41, 42). Foxn1nu mice were subcutaneously injected with A549 cells and treated with either 300 U/kg EPO (6, 25) or saline. EPO treatment did not increase the growth of A549 wt tumors (**Supplemental Figure 1A**) or alter cellular respiration (**Supplemental Figure 1B**), but it increased erythropoiesis as expected (**Supplemental Figure 1C**). Next, two A549 EPOR knockdown cell clones (shEPOR1+2) with the expected reduced pAKT levels (9) and two scrambled control cell clones (shSCR1+2) (**Supplemental Figure 2A**) were subcutaneously injected into Foxn1nu mice, which were treated with either EPO or saline. A549 shEPOR tumors had 5 times lower EPOR protein levels than shSCR tumors (**Figure 1A**). EPO treatment increased erythropoiesis in all mice (**Supplemental Figure 2B**) but did not increase tumor progression, weight, or volume (**Supplemental Figures 2C–F**). However, shSCR tumors grew faster than shEPOR tumors did. When shSCR1 tumors reached the maximum permitted tumor size (which led to the termination of the subcohort experiment), they were 4 times larger than the size of shSCR2, shEPOR1, and shEPOR2 tumors (p<0.05). 56 days after tumor cell injection, shSCR2 tumors were 4 times larger than shEPOR1 and shEPOR2 tumors (p<0.01) (**Figure 1B**), indicating that the loss of EPOR is associated with reduced tumor growth in A549 lung cancer cells.

### Knockdown of EPOR decreases cellular respiration of A549 lung cancer xenografts in Foxn1nu mice

**Figure 2:**
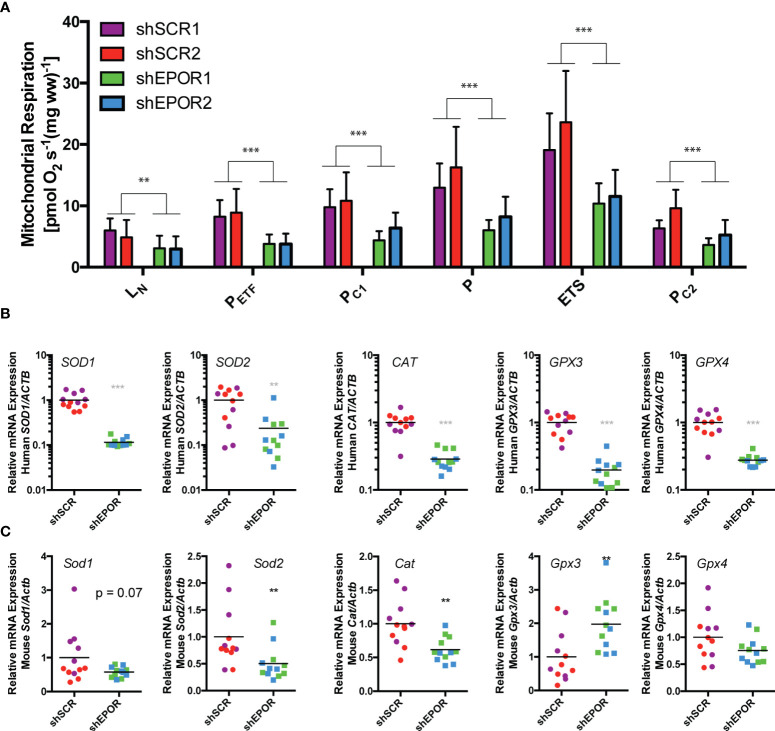
Knockdown of EPOR reduces cellular respiration of human A549 lung cancer xenografts in Foxn1nu mice. Biopsies of human A549 tumors that either express EPOR (shSCR1/2) or not (shEPOR1/2) were isolated from Foxn1nu mice and mass-specific respiration was immediately measured by high-resolution respirometry. Panel **(A)** shows the mass-specific respiration per unit weight of freshly isolated tumor biopsies of shSCR and shEPOR A549 tumors (n=6-7). L_N_, respiration in the absence of adenylates; P_ETF_, capacity for fatty acid β-oxidation; P_C1_, submaximal state 3 respiration through complex I; P, maximal state 3 respiration - oxidative phosphorylation capacity; ETS, electron transport system capacity; P_C2_, submaximal state 3 respiration through complex II. Relative mRNA expression of human and murine genes was analyzed by qPCR in A549 tumors: Shown are **(B)** mRNA levels of human superoxide dismutase 1 (*SOD1*), human superoxide dismutase 2 (*SOD2*), human catalase (*CAT*), human glutathione peroxidase 3 (*GPX3*) and human glutathione peroxidase 4 (*GPX4*) normalized to human b-Actin (*ACTB*) mRNA levels as well as **(C)** mRNA levels of murine superoxide dismutase 1 (*Sod1*), murine superoxide dismutase 2 (*Sod2*), murine catalase (*Cat*), murine glutathione peroxidase 3 (*Gpx3*) and (murine glutathione peroxidase 4 (*Gpx4*) normalized to murine b-Actin (*Actb*) mRNA levels of shSCR and shEPOR A549 tumors (n=16). Data are shown as means and standard deviations **(A)** or as scattered blots with mean and individual data distribution (**B, C**) of each control or EPOR-knockdown clone (control: shSCR1 purple, shSCR2 red; EPO- knockdown: shEPOR1 green and shEPOR2 blue tumor samples). The graphs in panel **(B)** are on a logarithmic scale. Data were analyzed by a Student’s t-test (black stars) or by a Mann-Whitney test (grey stars). ***p<0.001; **p<0.01.

Similar to tumor growth, EPO treatment did not alter cellular respiration (**Supplemental Figures 3A–D**) but the loss of EPOR reduced cellular respiration in A549 tumors. shEPOR tumors showed lower mass-specific respiration than shSCR tumors (**Figure 2A**): the mean mass-specific rates of respiration representing maximal fatty acid-fueled β-oxidation and electron input *via* electron-transferring flavoprotein (P_ETF_) of shEPOR tumors was 2.3 times lower than in shSCR tumors (p<0.01). The mean state 3 respiration driven by complex I-linked substrates (P_CI_) was 1.9 times lower (p<0.001) and the mean maximal state 3 respiration with electron input from mitochondrial complexes 1 and 2 (P) was 2.1 times lower (p<0.001) in shEPOR tumors than in shSCR tumors. The mean maximal electron transport system capacity (ETS) representing maximal non-coupled respiration from adenylate phosphorylation was 1.9 times lower (p<0.001), and the mean rate of state 3 respiration driven by complex II-linked substrates (P_CII_) was 1.8 times lower in shEPOR tumors than in shSCR tumors (p<0.001). We measured the mRNA expression of human and murine oxidative stress-related genes as an approximation for cellular respiration rates (43). The mean human mRNA levels of superoxide dismutase 1 and 2 (*SOD1* and *2*) in shEPOR tumors were 8.7 times (p<0.001) and 4.2 times lower (p<0.001) than in shSCR tumors. Additionally, catalase (*CAT*) and glutathione peroxidase 3 and 4 (*GPX3* and *4*) in shEPOR tumors were 3.5, 5, and 3.6 times lower than those in shSCR tumors (p<0.001) (**Figure 2B**), implying that the proportion of shEPOR A549 cancer cells in the tumor biopsies respire less than shSCR A549 cancer cells. The mean murine mRNA levels of *Sod1* in shEPOR tumors tended to be lower, and the *Sod2* mRNA levels were two times lower (p<0.01) than those in shSCR tumors. In addition, the *Cat* mRNA levels in shEPOR tumors were 1.6 times lower than those in shSCR tumors (p<0.01). Both the *Gpx3* and *Gpx4* mRNA levels were not reduced in shEPOR tumors, while the mean *Gpx3* mRNA levels in shEPOR tumors were two times higher than those in shSCR tumors (p<0.01) (**Figure 2C**). This observation revealed that respiratory control and reciprocal cellular antioxidant capacity were predominantly reduced in human-derived shEPOR cancer cells, and to a lesser extent, in the adjacent murine stromal cells of shEPOR tumors.

### Knockdown of EPOR decreases mitochondrial content in A549 lung cancer xenografts in Foxn1nu mice

**Figure 3:**
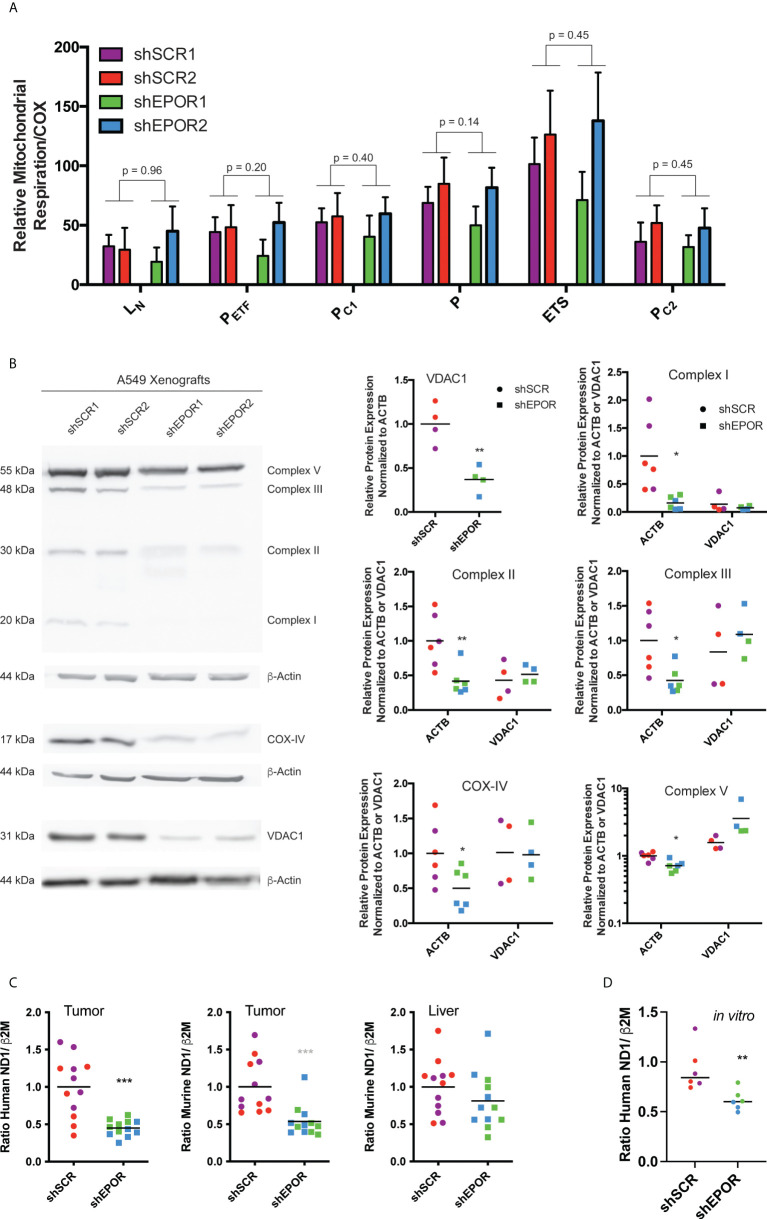
Knockdown of EPOR reduces mitochondrial content of human A549 lung cancer xenografts in Foxn1nu mice. Biopsies of human A549 tumors expressing either EPOR (shSCR1/2) or not (shEPOR1/2) were isolated from Foxn1nu mice, and mitochondria-specific respiration was measured by high-resolution respirometry. Panel **(A)** shows mitochondria-specific respiration normalized to cytochrome c oxidase (COX) activity in freshly isolated shSCR and shEPOR A549 tumor biopsies (n=6-7). L_N_, respiration in the absence of adenylates; P_ETF_, capacity for fatty acid β-oxidation; P_C1_, submaximal state 3 respiration through complex I; P, maximal state 3 respiration - oxidative phosphorylation capacity; ETS, electron transport system capacity; P_C2_, submaximal state 3 respiration through complex II. Panel **(B)** shows a representative western blot image of specific subunits from complexes of the oxidative phosphorylation (OXPHOS) from control tumors (shSCR) and EPOR-knockdown tumors (shEPOR) by using an anti-total OXPHOS antibody cocktail: Complex V: 55 kDa (ATP5A, ATP synthase mitochondrial F1 complex alpha 1); Complex III: 48 kDa (UQCRC2, cytochrome b-c1 complex subunit 2); Complex II: 30 kDa (SDHB, succinate dehydrogenase [ubiquinone] iron-sulfur subunit); Complex I: 20 kDa (NDUFB8, NADH dehydrogenase [ubiquinone] 1 beta subcomplex subunit 8). Complex IV: 17 kDa was visualized using an anti-cytochrome c oxidase antibody. VDAC1: 31 kDa (voltage-dependent anion-selective channel 1) was used as a mitochondrial marker independent of OXPHOS complexes and β-actin 44 kDa was used as a loading control. The band intensity of proteins after western blotting was quantified using MCID Analysis 7.0 and normalized either to β-actin to estimate expression levels per cell, or to VDAC1 to normalize the protein levels to mitochondrial content. Relative protein expression levels of VDAC1, complex I, complex II, complex (III), COX-IV (complex IV), and complex V are shown for control (shSCR) and EPOR-knockdown (shEPOR) tumors (n=4-6). **(C)** Mitochondrial content was determined by the ratio of human (left panel) or murine (middle and right panel) MT-ND1 (mitochondrially encoded NADH dehydrogenase 1) mitochondrial DNA to human or murine β2M (β-2microglobulin) genomic DNA, respectively, which were quantified by qPCR from DNA extracts of A549 control (shSCR) and EPOR-knockdown (shEPOR) tumors or the liver (right panel) (n=12). **(D)** Likewise, mitochondrial content of *in vitro* cultured shSCR and shEPOR clones was determined by the ratio of human MT-ND1 to human β2M genomic DNA. Data are presented as **(A)** mean and standard deviation or as scattered blot with mean and individual data distribution for each clone (shSCR1 purple, shSCR2 red, shEPOR1 green, and shEPOR2 blue tumor samples). Data were analyzed by a Student’s t-test (black p-values; stars) or by a Mann-Whitney test (grey stars). ***p<0.001; **p<0.01; *p<0.05.

Next, we showed that the mitochondrial content is reduced, while respiratory rates per unit mitochondria remain unaffected in shEPOR tumors. First, we analyzed mitochondria-specific respiration by normalizing mass-specific respiratory rates to cytochrome c oxidase (COX) activity (29, 44). We observed no differences in mitochondria-specific respiration between the shSCR and shEPOR tumors (**Figure 3A**). Additionally, the slight difference in the mRNA levels of genes regulating mitochondrial fusion or fission did not seem to account for the difference in mass-specific respiration between shEPOR and shSCR tumors (**Supplemental Figure 4**). The mean protein expression of the mitochondria-specific biomarker voltage-dependent anion-selective channel 1 (VDAC1) was 2.7 times lower in shEPOR than in shSCR tumors. Similarly, the protein expression of OXPHOS markers in shEPOR tumors was lower than that in shSCR tumors (**Figure 3B**). The mean expression of complex I (NDUFB8) was 6.2 times lower (p<0.05), mitochondrial complex II (SDHB) expression and complex III (UQCRC2) expression were both 2.4 times lower (p<0.01 and p<0.05, respectively), complex IV (COX-IV) expression was 2 times lower (p<0.05), and complex V (ATP5A) expression was 1.4 times lower (p<0.01) in shEPOR than in shSCR tumors. When normalizing protein expression to the OXPHOS-independent mitochondrial marker VDAC1, no differences in expression levels between shSCR and shEPOR tumors were detected (**Figure 3B**). Thus, the prevalence of cellular mitochondria was reduced in shEPOR tumors, while respiratory rates per unit mitochondria were unaffected. The relative amounts of human mitochondrial mtDNA in shEPOR tumors (**Figure 3C**) as well as in *in vitro* cultured shEPOR cells (**Figure 3D**) were ~2 times lower (p<0.001) and ~1.5 times lower (p<0.01) than those in shSCR tumors and cells, respectively. Also, the murine mitochondrial mtDNA in shEPOR tumors was ~2 times lower than in shSCR tumors (p<0.001), confirming that both human A549 shEPOR cancer cells and adjacent murine stromal cells in EPOR-deficient tumors had fewer mitochondria than in shSCR tumors. Murine mtDNA content in the liver of mice with A549 shSCR or shEPOR tumors was essentially similar (**Figure 3C**), implying that the reduced mitochondrial content is restricted to the respective surrounding tumor and its microenvironment.

### Knockdown of EPOR is associated with impaired iNOS expression in A549 lung cancer xenografts in Foxn1nu mice

**Figure 4:**
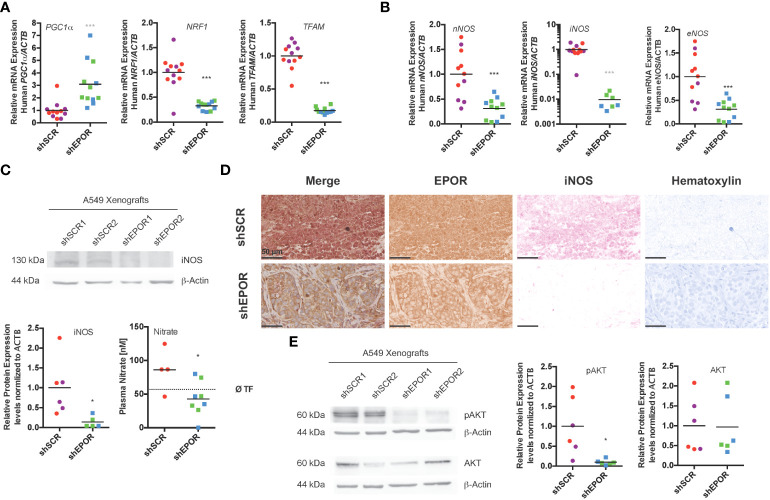
Knockdown of EPOR impairs iNOS expression and AKT phosphorylation in A549 lung cancer xenografts in Foxn1nu mice. Biopsies of human A549 tumors expressing EPOR (shSCR1/2) or not (shEPOR1/2) were isolated from Foxn1nu mice, and levels of key mitochondrial biogenesis, as well as nitric oxide synthesis genes and proteins, were quantified by qPCR and western blotting. **(A)** Shown are the human mRNA levels of mitochondrial biogenesis genes peroxisome proliferative activated receptor, gamma, coactivator 1α (*PGC-1a*), nuclear respiratory factor 1 (*NRF1*) and transcription factor A, mitochondrial (*TFAM*) quantified by qPCR and normalized to β-actin (*ACTB*) mRNA expression levels (n=12). Panel **(B)** shows the mRNA levels of nitric oxide synthase genes *nNOS, iNOS*, and e*NOS* from control (shSCR) and EPOR-knockdown (shEPOR) tumors quantified by qPCR and normalized to β-actin (*ACTB*) mRNA (n=6-12). Notably, iNOS mRNA was not detectable in five samples (two from clone shEPOR1 and three from shEPOR2), and the scale is logarithmic. Panel **(C)** shows representative western blot images of iNOS (130 kDa) from protein extracts of control tumors (shSCR) and EPOR-knockdown tumors (shEPOR) (n=3). β-actin (44 kDa) was used as a loading control. The band intensities of proteins after western blotting images were quantified using MCID Analysis 7.0 and normalized to β-actin. The relative protein expression levels of iNOS are shown for control (shSCR) and EPOR- knockdown (shEPOR) tumors (n=6). Furthermore, the plasma nitrate values of mice with shSCR and shEPOR tumors are shown (right panel). The dotted black line indicates the reference value for three tumor-free Foxn1nu mice (n=4-7). **(D)** Tumor sections of control (shSCR1) and EPOR-knockdown (shEPOR1) tumors were immunohistochemically stained for EPOR (brown) and iNOS (pink) and counterstained with hematoxylin (blue). Panel **(E)** shows a representative western blot image of phospho-AKT (60 kDa) and AKT (60 kDa) from protein extracts of control tumors (shSCR) and EPOR-knockdown tumors (shEPOR) (n=3). β-actin (44 kDa) was used as a loading control. The band intensity of proteins on western blotting images was quantified using MCID Analysis 7.0 and normalized to β-actin. Relative protein expression levels of pAKT and AKT are shown for control (shSCR) and EPOR-knockdown (shEPOR) tumors (n=6). Data are presented as scattered blots with the mean and individual data distribution of each clone (shSCR1 purple, shSCR2 red, shEPOR1 green, and shEPOR2 blue tumor samples). Data were analyzed by a Student’s t-test (black stars) or by a Mann-Whitney test (grey stars). ***p<0.001; *p<0.05.

We tested whether fewer mitochondria in shEPOR A549 tumors resulted from a blunted signal for mitochondrial biogenesis. We measured the mRNA expression of transcriptional regulators involved in mitochondrial biogenesis, *PGC1a*, *NRF1*, and *TFAM* (45–47). The mean levels of *PGC1a* mRNA in shEPOR tumors were 3 times higher than those in shSCR tumors (p<0.001), while the mean levels of *NRF1* and *TFAM* mRNA in shEPOR tumors were 3 and 5.8 times lower than in shSCR tumors (p<0.001), suggesting that transcription to realize mitochondrial biogenesis was reduced in shEPOR tumors (**Figure 4A**). Murine mRNA levels of *Pgc1a*, *Nrf1*, and *Tfam* did not differ between shSCR and shEPOR tumors (**Supplemental Figure 5A**). We speculated that reduced mitochondrial biogenesis resulted from impaired NO synthesis; thus, we analyzed the mRNA expression of all three nitric oxide synthase isoforms. The mean mRNA levels of *nNOS* and *eNOS* in shEPOR tumors were reduced by factor 3.2 (p<0.001) and factor 4.2 (p<0.001), respectively. Interestingly, *iNOS* mRNA expression was detected in all shSCR tumors, but only in 7 out of 12 shEPOR tumors. In these tumors, *iNOS* mRNA levels were 100 times lower than those in shSCR tumors (p<0.001) (**Figure 4B**). Mean murine *iNos* mRNA levels in murine stromal cells were only slightly reduced, with no change in *eNos* mRNA levels (**Supplemental Figure 5A**). Moreover, the mean iNOS protein levels in shEPOR tumors were 7.3 times lower than those in shSCR tumors (p<0.05) (**Figure 4C**). Additionally, plasma nitrate was assessed (nitrite was not detected in plasma samples), as an indirect measure of NO concentration in tumor-bearing mice. Nitrate did not correlate with tumor size (R^2 =^ 0.154; p=0.21), suggesting that tumor size was not a major predictor of plasma nitrate levels. However, the nitrate levels in mice with shEPOR tumors were two times lower than those in mice with shSCR tumors (p<0.05), implying that low iNOS expression levels in shEPOR tumors indeed result in lower NO production and, in turn, impaired mitochondrial biogenesis (**Figure 4C**). When we immunohistochemically analyzed EPOR and iNOS protein expression in shSCR and shEPOR tumor sections, we observed that both proteins were downregulated in shEPOR tumors (**Figure 4D**). To test whether the EPOR-dependent effect on mitochondrial biogenesis requires iNOS, we analyzed mRNA, protein, and gDNA samples isolated from paraffin-embedded, MDA-MB-231 breast cancer xenografts that were produced and analyzed in a previous study (5), and that did or did not express EPOR. MDA-MB-231 breast cancer xenografts did not express detectable amounts of iNOS mRNA. The loss of EPOR in these iNOS-deficient tumors did not alter the cellular signaling for mitochondrial biogenesis genes (**Supplemental Figure 5B**), suggesting that iNOS is required to control mitochondrial biogenesis downstream of EPOR. However, rescuing iNOS expression alone in shEPOR A549 tumors did not increase *TFAM* or *NRF1* expression (**Supplemental Figure 5C**), suggesting that additional co-factors are required to mediate this effect. To control mitochondrial biogenesis in muscle cells, iNOS acts in concert with AKT to activate NRF-1 and TFAM (16). Indeed, pAKT levels were 10 times lower in shEPOR tumors than in shSCR tumors (p<0.05), whereas total AKT levels did not differ (**Figure 4E**).

### iNOS and pAKT together regulate mitochondrial biogenesis downstream of EPOR

**Figure 5:**
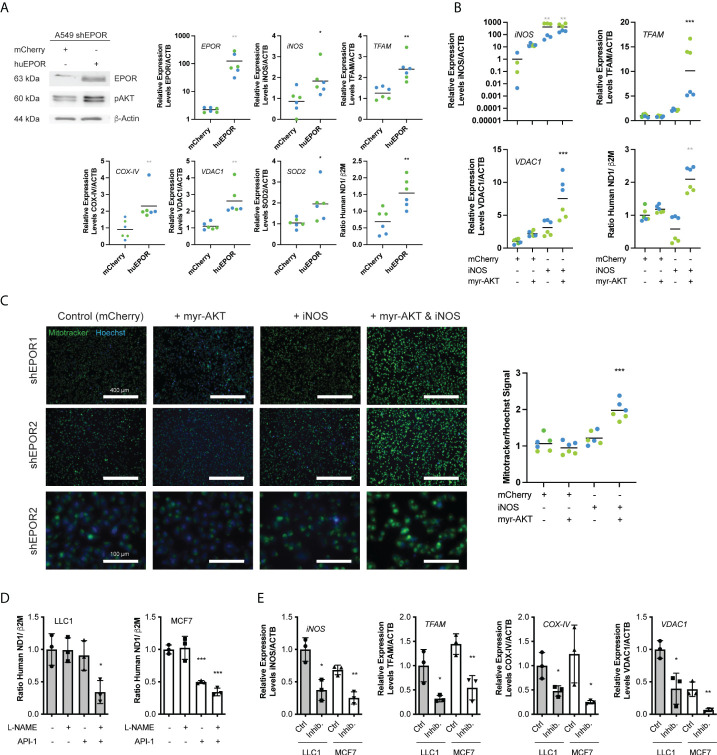
iNOS and AKT are required to mediate the EPOR effect on mitochondrial biogenesis. A549 shEPOR1 (green symbols) and shEPOR2 (blue symbols), LLC1 murine Lewis lung carcinoma cells (grey bars), and human MCF-7 breast cancer cells (white bars) were cultivated *in vitro*. **(A)** EPOR-knockdown shEPOR1 A549 cells were transfected with huEPOR or a control (mCherry) plasmid, and 72 h after transfection, mRNA and protein were isolated. Shown is a representative western blot image of human erythropoietin receptor (EPOR) (63 kDa), pAKT (60 kDa), and loading control β-actin (44 kDa) (left panel), as well as mRNA levels of erythropoietin receptor (*EPOR*), inducible nitric oxide synthase (*iNOS*), transcription factor A, mitochondrial (*TFAM*), cytochrome c oxidase subunit 4.2 (*COX-IV*), voltage-dependent anion-selective channel 1 (*VDAC1*), and superoxide dismutase 2 (*SOD2*) quantified by qPCR and normalized to β-actin (*ACTB*) mRNA (n=6). Further shown is mitochondria content (right panel) determined by the ratio of human MT-ND1 (mitochondrially encoded NADH dehydrogenase 1) mitochondrial DNA to β2M (β-2microglobulin) genomic DNA, which was quantified by qPCR from genomic DNA extracts. **(B)** EPOR-knockdown shEPOR1 and 2 A549 cells were incubated with lentiviral vectors to stably express inducible nitric oxide synthase (iNOS) or mCherry (control). Additionally, cells were transfected with a plasmid to overexpress constitutively active myr-AKT (24). The cells were incubated for 72 h, and mRNA was isolated. *iNOS*, *TFAM*, and *VDAC1* mRNA levels were quantified using qPCR and normalized to *ACTB.* Furthermore, the mitochondria content (right lower panel) was determined by the ratio of human MT-ND1 (mitochondrially encoded NADH dehydrogenase 1) mitochondrial DNA to β2M (β-2microglobulin) genomic DNA, which was quantified by qPCR from genomic DNA extracts (n=6). **(C)** Shown are images of shEPOR1 (upper row) and shEPOR2 (middle and bottom rows) of A549 cells stably expressing iNOS or mCherry (control) and were transfected with a plasmid to myr-AKT or not. Cells were incubated with Mitotracker (green) and Hoechst (blue), and images were taken using a fluorescence microscope and quantified using ImageJ. Shown in the right panel is the Mitotracker signal normalized to the Hoechst signal (n=4-6). **(D)** Murine LLC1 (grey bars) and human MCF-7 cells (white bars) were incubated with 200 µM L-NAME and/or 5 µM API-1 for 72 h. Mitochondrial content was determined by the ratio of murine (LLC1) or human (MCF7) MT-ND1 (mitochondrially encoded NADH dehydrogenase 1) mitochondrial DNA to murine or human β2M (β-2microglobulin) genomic DNA, which was quantified by qPCR from genomic DNA extracts (n=3). **(E)** Further shown are RNA levels of *iNOS*, *TFAM, COX-IV*, and *VDAC1* quantified by qPCR and normalized to *ACTB* from LLC1 and MCF-7 cells either treated *in vitro* for 72 h with 200 µM L-NAME + 5 µM API-1 (Inhib.) to simultaneously inhibit iNOS and AKT or not (Ctrl.) (n=3). Data are shown as scattered blots with mean and individual data distribution of each clone (shEPOR1 green and shEPOR2 blue) or as bars with scatter dot plots (LLC1 grey and MCF-7 white). Data were analyzed by a Student’s t-test (black stars), a Mann-Whitney test (grey stars), an one-way ANOVA with Bonferroni *post hoc* test (black stars), or a Kruskal Wallis test with Dunn’s multiple comparison test (grey stars) (***p<0.001; **<0.01; *p<0.05).

Similar to tumors, *in vitro* cultured shEPOR cells showed reduced expression levels of *iNOS*, *PGGC1a*, *NRF1*, and *TFAM* (**Supplemental Figure 6A**). To identify the mechanism that regulates mitochondrial biogenesis downstream of EPOR, we transiently rescued EPOR expression in shEPOR1/2 knockdown cells. Cells re-expressing huEPOR showed 100 times higher EPOR mRNA levels than control-transfected cells (p<0.01) as well as increased EPOR and pAKT protein levels. EPOR re-expression was associated with increased expression of *iNOS* (2.1 times, p<0.05), *TFAM* (2.5 times, p<0.01), *COX-IV* (2.5 times, p<0.01), *VDAC1* (2.4 times, p<0.01) and a 2.2 times higher ratio of mtDNA to gDNA (**Figure 5A**), whereas the expression of *nNOS*, *eNOS*, *NRF1*, and *PGC1α* did not change (**Supplemental Figure 6B**).shEPOR1/2 cells stably expressing iNOS showed 420 times higher *iNOS* mRNA levels than control cells (p<0.001) but did not significantly increase *TFAM* and *VDAC1* mRNA levels or alter mitochondrial content when assessed by the mtDNA/gDNA ratio (**Figure 5B**) or by Mitotracker (**Figure 5C**). Likewise, the transient overexpression of constitutively active myr-AKT (24) alone (**Supplemental Figure 6C**) was not sufficient to stimulate mitochondrial biogenesis (**Figures 5B**, **C**). However, the co-expression of iNOS and myr-AKT increased *TFAM* and *VDAC1* mRNA levels 10 times (p<0.001) and 7.5 times (p<0.001), respectively (**Figure 5B**). Likewise, the mitochondrial content increased approximately 2 times when assessed by the ratio of mtDNA/gDNA (p<0.01) (**Figure 5B**) or by Mitotracker (p<0.001) (**Figure 5C**). To test whether iNOS and AKT regulate mitochondrial biogenesis in other cancer cells, we used iNOS-expressing LLC1 and MCF7 cells and treated them with the iNOS inhibitor L-NAME and the AKT inhibitor (API-1). While the inhibition of either iNOS or AKT by L-NAME or API-1 did not reduce mitochondrial content in LLC1 cells, the combination of both inhibitors reduced the mtDNA/gDNA ratio by 65% (p<0.05). In MCF7 cells, iNOS inhibition was not sufficient to reduce cellular mitochondria, whereas the inhibition of AKT by API-1 was sufficient to lower the mitochondrial content by 50% (p<0.001). The double inhibition of iNOS and AKT, lowered the mitochondrial content by 65% (p<0.001) (**Figure 5D**). Reduced mitochondrial content in cells treated with both inhibitors (L-NAME and API-1) was associated with lower *iNOS*, *TFAM*, *COX-IV*, and *VDAC1* mRNA levels (**Figure 5E**), while the expression of SOD1 and NRF1 was not significantly different (**Supplemental Figure 6D**). In summary, our data indicate that downstream of EPOR, both iNOS and AKT are required to control mitochondrial biogenesis.

### EPOR expression correlates with the mitochondrial marker VDAC1 in biopsies of human lung cancer patients

**Figure 6:**
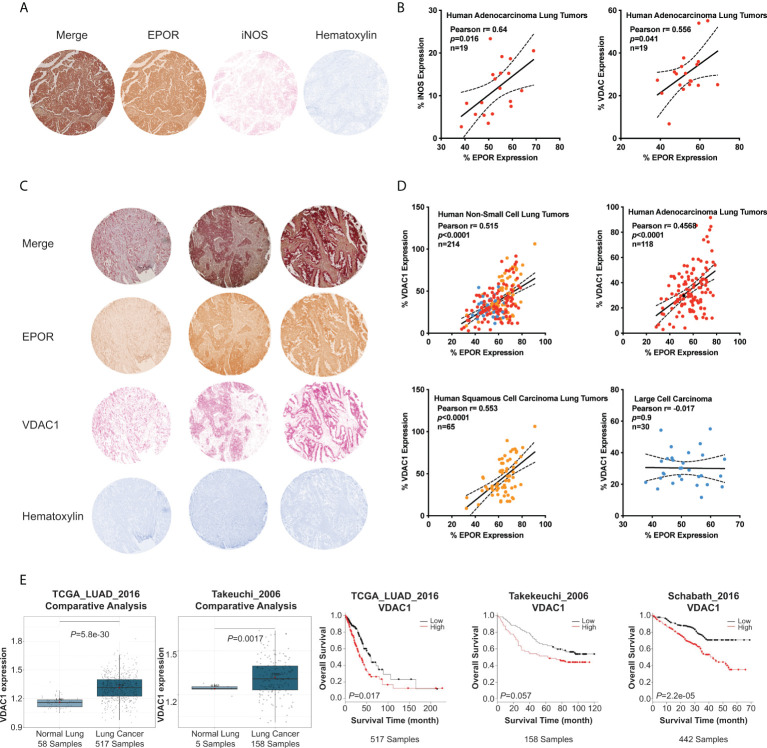
EPOR expression correlates with VDAC1 expression in human lung cancer biopsies. Human non-small lung cancer tissue arrays were immunohistochemically stained for EPOR, VDAC1, and iNOS. **(A)** Shown is a representative tumor core image stained for EPOR (brown), iNOS (red), and counterstained with hematoxylin (blue). **(B)** EPOR and iNOS expression (left panel), as well as EPOR and VDAC1 expression (right panel), were quantified by ImageJ and normalized to the total measured tumor core area. Shown are Pearson correlation analyses of normalized EPOR (x-axis) and iNOS and VDAC1, respectively (y-axis) expression levels (in %) from tumor core images of human lung adenocarcinoma (n=19). **(C)** Shown are three representative tumor core images stained for EPOR (brown), VDAC1 (red), and counterstained with hematoxylin (blue). **(D)** EPOR and VDAC1 expression (i.e., stained area) were quantified by ImageJ and normalized to the total measured tumor core area. Shown are Pearson correlation analyses of normalized EPOR (x-axis) and VDAC1 (y-axis) expression levels (in %) from all tumor core images (upper left panel; n=214) or tumor core images of human adenocarcinoma lung tumors (upper right panel; red; n=118), human squamous cell carcinoma lung tumors (lower left panel; orange; n=65) and human large cell carcinoma (lower right panel; blue; n=30). **(E)** Analyses of *VDAC1* mRNA expression in lung adenocarcinoma patients using the lung cancer explorer (37). The first and second panels show *VDAC1* levels in normal lungs and lung adenocarcinoma in the TCGA_LUAD_2016 study (1^st^ panel) (38) and from Takeuchi_2006 study (2^nd^ panel) (39). In panels 3-5, Kaplan-Meier survival curves show an association between overall survival of lung adenocarcinoma patients and the mRNA expression of *VDAC1* in the TCGA_LUAD_2016 study (3^rd^ panel) (38), the Takeuchi_2006 study (4^th^ panel) (39), and the Schabath_2016 study (5^th^ panel) (40). The datasets were split into low and high VDAC1 expression by using the overall mean of VDAC1 expression and were analyzed with a log-rank test.

To validate whether EPOR contributes to the regulation of mitochondrial biogenesis in human lung cancer patients, we analyzed EPOR, iNOS, and VDAC1 expression in lung adenocarcinoma tissue from 19 human patients using immunohistochemistry (**Figure 6A**). We observed that both VDAC1 (Pearson’s r = 0.556, p<0.041) as well as iNOS (Pearson’s r = 0.64, p<0.016) correlated with EPOR expression and concluded that EPOR-expressing lung cancer cells showed an increased expression of iNOS and the mitochondrial marker, VDAC1 (**Figure 6B**). We then validated these findings by analyzing EPOR and VDAC1 expression in arrays of non-small lung cancer tissue from 214 human patients (**Figure 6C**). Across all tumor sections, EPOR expression predicted VDAC1 expression (Pearson’s r = 0.515, p<0.0001) (**Figure 6D**). When we analyzed biopsies of lung cancer patient subgroups, we found that EPOR positively correlated with VDAC1 expression in human adenocarcinoma lung tumors (Pearson’s r = 0.4568, p<0.0001) as well as in human squamous cell carcinoma lung tumors (Pearson’s r = 0.553, p<0.0001), while no correlation was found in human large cell carcinoma biopsies (**Figure 6D**). Next, we analyzed lung adenocarcinoma datasets (38–40) using the lung cancer explorer (37) (**Figure 6E**). *VDAC1* mRNA levels are higher in lung adenocarcinoma than in healthy lung tissue. Although the Takeuchi_2006 dataset only included five samples of normal lung tissue, *VDAC1* levels in these five samples cluster at the lower 25% percentile of lung adenocarcinoma. Furthermore, we observed that *VDAC1* expression, as a marker for mitochondrial content, is associated with reduced survival in three lung adenocarcinoma datasets. Because our data provide convincing evidence that EPOR supports mitochondrial biogenesis in patients with lung cancer, it may be a target to control mitochondrial content and cancer metabolism.

## Discussion

In this study, we asked whether EPO/EPOR controls mitochondrial and concomitant cellular metabolism in malignant tissues *via* its receptor EPOR. Although EPO treatment of wild-type A549 tumor-bearing mice did not alter tumor growth or respiratory control, the loss of EPOR *per se* reduced tumor growth and mitochondrial density with an unabated respiratory potential. Both human cancer and murine stromal cells comprising the tumor expressed fewer mitochondria, indicating that the loss of EPOR in tumor cells affects the whole tumor microenvironment. We suspected that NO controls mitochondrial biogenesis in the tumor microenvironment and showed that iNOS expression (and thus, NO production) is a key signaling agent that regulates mitochondrial biogenesis *via* the EPOR in A549 tumors. In addition to iNOS expression, AKT activation was also involved in controlling mitochondrial biogenesis. The absence of either iNOS or pAKT is sufficient to inhibit the EPOR-specific regulation of mitochondrial biogenesis, indicating that AKT and iNOS collectively regulate mitochondrial biogenesis downstream of EPOR in lung cancer cells. Finally, EPOR expression and the expression of the mitochondrial marker VDAC1 are positively correlated in biopsies of human non-small lung cancer patients, suggesting that the herein-reported mechanisms exist in tumors of human (lung) cancer patients.

### EPOR knockdown reduces A549 tumor growth

While EPO has been shown to induce progression and survival in different cancer cells (5, 48, 49), EPO treatment in our study did not increase the growth of wild-type A549 xenografts. Such non-responsiveness to EPO has been previously observed in EPOR-expressing A549 cells (42) as well as in some breast cancer cell lines (9). Interestingly, the loss of EPOR in A549 lung cancer cells reduced the proliferation of tumor xenografts, which has been also observed e.g., in glioma cells (12), implying that EPOR *per se* has a regulatory role in cancer cells. It is currently unknown whether EPOR in cancer cells exists as a homodimer or as a heterodimer [with the common-β receptor subunit (CD131)] with a much lower EPO affinity (50, 51), whether EPOR activation is ligand-independent (52), or whether endogenously produced EPO, either by the kidney or by the tumor itself (2, 10, 53), is sufficient to fully activate EPOR in A549 tumors. The implication that endogenous EPO may be sufficient to support tumor growth suggests that targeting EPOR on tumor cells is a relevant approach to attenuate tumor growth while enabling treatment with EPO to alleviate anemia.

### EPOR regulates mitochondrial biogenesis through iNOS and pAKT in A549 lung tumors

Loss of EPOR in A549 tumors led to reduced mass-specific respiration rates, while mitochondria-specific respiration and OXPHOS protein expression levels per mitochondrion did not differ between EPOR-deficient and EPOR-expressing tumors, indicating that the respiratory capacity per mitochondrial unit was not affected in EPOR-deficient tumors. However, high-resolution respirometry does not allow to differentiate between the respiratory potentials of distinct cell types (e.g., human cancer and murine stromal cells in the current study) in a heterogeneous tumor sample. Therefore, we used human lung cancer cells to grow tumors in immunocompromised Foxn1nu mice, which enabled us to differentiate the gene expression and mitochondrial DNA levels between human cancer cells and murine stromal cells. Indeed, the mitochondrial content was diminished in shEPOR tumors. The downregulation of the transcription factor NRF1 and TFAM suggests that transcription to realize mitochondrial biogenesis was impaired (45, 46). In contrast to NRF1 and TFAM, mRNA levels of PGC1 α, representing a key regulator of mitochondrial metabolism (47), were elevated in EPOR deficient tumors. In contrast to our study, the lack of eNOS and AKT also reduced PGC1α levels in muscle cells (16). PGC1 α is critical for cellular energy management (54) and disturbed energy metabolism in EPOR deficient tumors possibly led to an over-compensatory expression of PGC1 α, which has been described in brown adipose tissue with mitochondrial dysfunction (55). Mitophagy (data not shown) or mitochondrial fusion and fission did not significantly contribute to the reduced mitochondrial content in A549 tumors. Thus, we concluded that EPOR-deficient tumors had a lower mitochondrial density, mainly due to impaired mitochondrial production.

Interestingly, murine stromal cells in EPOR-deficient A549 tumors also had fewer mitochondria than those in the control tumors, suggesting that tumor EPOR controls mitochondria on a cell-by-cell basis as well as in a paracrine fashion. This effect was mainly restricted to the tumor microenvironment because the mitochondrial DNA levels in the liver of mice carrying either EPOR-deficient or EPOR-expressing A549 tumors did not differ. We asked whether this local effect is controlled by EPOR-dependent NO production because NO has been reported to be induced by EPO (56–58) and to activate NRF-1 mediated mitochondrial biogenesis (16). Indeed, EPOR-deficient tumors showed reduced iNOS mRNA levels, associated with reduced plasma nitrate concentrations, which were measured as an indirect approximation of plasma NO levels. When we analyzed mitochondrial content in iNOS-deficient MDA-MB-231 breast cancer tumors (59) from a previous study (5), the loss of EPOR in these tumors did not influence mitochondrial content or transcriptional regulators of mitochondrial biogenesis, suggesting that iNOS expression is essential for EPOR-dependent control of mitochondrial biogenesis. However, rescuing iNOS expression in EPOR-deficient A549 cells and tumors was not sufficient to increase mitochondrial content suggesting that the EPOR-dependent control of mitochondrial biogenesis requires iNOS, among other factors. Previous studies have suggested that controlling mitochondrial biogenesis *via* EPO requires both, eNOS and pAKT (16). EPOR-deficient A549 tumors had lower pAKT levels than control tumors and AKT is often phosphorylated by EPO/EPOR in other (cancer) cells, supporting their growth (5, 9) and regulating mitochondrial biogenesis (60). Indeed, when we rescued iNOS and pAKT levels simultaneously in EPOR-deficient A549 cells, *TFAM* and *VDAC1* expression, as well as mitochondrial content, increased. The co-inhibition of iNOS and AKT reduced mitochondrial density and TFAM in additional lung and breast cancer cell lines, suggesting that other cancer cells also rely on the regulation of mitochondrial biogenesis by iNOS and pAKT downstream of EPOR.

Our *in vitro* and *in vivo* data were supported by a positive correlation of EPOR and iNOS, analyzed in tissue arrays of human non-small lung cancer patients. Furthermore, EPOR and VDAC1, as a surrogate of mitochondrial content (61) correlated positively in most lung cancer types, except for large cell carcinoma. Although VDAC1 is also differentially expressed by apoptotic regulation (62), we propose that the approximately 27% (r^2^ squared Pearson) of VDAC1 variation among the lung cancer biopsies, which was explained by EPOR expression, reflects differences in mitochondrial content (44, 61, 63, 64). When we analyzed VDAC1 expression levels in different datasets of lung adenocarcinomas (37–40), we observed that it is higher expressed in tumors than in healthy tissue and is associated with poor survival. This indicates that our results translate from preclinical research to human (lung) cancer patients and that targeting EPOR specifically in cancer cells may provide a new approach to control the expression of mitochondria in cancer cells and thus, tumor metabolism.

### Conclusion

We provide evidence that EPOR contributes to the regulation of mitochondria in cancer cells. EPOR controls the phosphorylation of AKT as well as the expression of iNOS and thus, NO production. In turn, pAKT and iNOS (through NO) regulate mitochondrial biogenesis in cancer and stromal cells. Our study suggests that an approach that solely targets EPOR in cancer cells may help control tumor metabolism and thereby the malignancy of tumors in human patients. Moreover, EPOR expression *per se* may also be a clinical predictor of cancer cell responsiveness to drugs and radiation, which depends on mitochondrial metabolism.

## Data availability statement

The original contributions presented in the study are included in the article/**Supplementary Material**. Further inquiries can be directed to the corresponding author.

## Ethics statement

The animal study was reviewed and approved by Veterinäramt Kanton Zürich, Zollstrasse 20, 8090 Zürich.

## Author contributions

MG, DN, and JAra initiated this project and MT further developed it. MA performed all cell biological experiments, analyzed, and interpreted the data, contributed to designing experiments, supported animal experiments, provided intellectual input, and contributed to writing the manuscript. FG performed immunohistochemical staining. NB, JArm, and HA supported animal experiments, sampling as well as sample analyses. MR prepared tissue samples and evaluated tumor sections. FM-R and JAra generated and provided A549 EPOR knockdown cells. AS performed statistical analyses. RJ and ES supported measurements of cellular respiration and gave intellectual input. TR contributed materials and analysis tools, DN, JAra, and MG provided intellectual input and contributed to writing the manuscript. MT designed experiments, conducted animal experiments, measured cellular respiration, analyzed, and interpreted the data, and wrote the manuscript. All authors contributed to the article and approved the submitted version.

## Funding

We acknowledge the financial support of the Swiss National Science Foundation (grant number 31003A_175637), Marie-Louise von Muralt Foundation, Krebsliga Switzerland (grant number KFS-3692-08-2015), J & F Thoma Foundation, the Forschungskredit of the University of Zurich, and the Zurich Center for Integrative Human Physiology (ZIHP). Furthermore, this study was supported by the FP7‐Health European Commission EpoCan grant (282551), and we kindly thank all members of the EpoCan consortium for discussions and support.

## Acknowledgments

The authors kindly thank Nicole Kachappilly, Nikolai Bogdanov, and the Center for Clinical Studies at the University of Zurich for their excellent technical support.

## Conflict of interest

The authors declare that the research was conducted in the absence of any commercial or financial relationships that could be construed as a potential conflict of interest.

## Publisher’s note

All claims expressed in this article are solely those of the authors and do not necessarily represent those of their affiliated organizations, or those of the publisher, the editors and the reviewers. Any product that may be evaluated in this article, or claim that may be made by its manufacturer, is not guaranteed or endorsed by the publisher.
