# Supplementary material for: Erythropoietin receptor regulates tumor mitochondrial biogenesis through iNOS and pAKT
Source: Front Oncol. 2022 Aug 16;12:976961. doi: 10.3389/fonc.2022.976961 (PMC9425774; doi:10.3389/fonc.2022.976961)
Supplement: Supplementary file 1 [file DataSheet_1.pdf]

# Supplementary Material

## *Erythropoietin receptor regulates tumor mitochondrial biogenesis through iNOS and pAKT*

## 1 Methods

*Animal handling and study design*

We used shEPOR1 cells that stably expressed either mCherry (control) or iNOS after lentiviral transduction and selection by the neomycin analog G-418. We injected control and iNOS expressing cells into each 8 Foxn1nu nude mice. Mice were constantly monitored (behavior, habitus) and body weight was recorded every other day. Tumor size was measured with a caliper and tumor volume was calculated by the formula V=1/2 x Length x $(Width)^2$ with the length being the largest tumor diameter and width the perpendicular tumor diameter (1).

*High-resolution respirometry*

Protocols for cellular respiration of tumor tissue were adopted from previous studies (2, 3). All chemicals were obtained from Sigma-Aldrich (Switzerland) Briefly, fresh tumor biopsy mass was collected (wet weight, mg) and kept in mitochondrial respiration buffer Miro05 (0.5mM EGTA, 3mM MgCl2•6H2O, 60mM K-lactobionate, 20mM taurine, 10mM KH2PO4, 20mM HEPES, 110mM sucrose, and 1g/L bovine serum albumin (pH 7.1) (2) on ice. Mitochondrial respiration of tissue was measured in mitochondrial respiration buffer Miro06 (Miro05 + 280 iU/ml catalase) at 37°C using the high-resolution Oxygraph-2k (Oroboros, Innsbruck, Austria). The instrument was calibrated to correct for back diffusion of oxygen into the chamber from the various components, leak from the exterior, oxygen consumption by the chemical medium, and sensor oxygen consumption. Oxygen flux was resolved by software allowing nonlinear changes in the negative time derivative of the oxygen concentration signal (Oxygraph-2k, Oroboros). All experiments were carried out in a hyper-oxygenated environment to prevent any potential oxygen diffusion limitation. To measure mass-specific respiration all parameters were normalized to the wet weight of the tissue biopsies. LN: Leak respiration (resting oxygen consumption of an unaltered and intact electron transport system in absence of adenylates) was measured after the addition of 2 mM malate and 0.2 mM octanoyl carnitine. PETF: Fatty acid oxidative capacity through electron-transferring flavoprotein (ETF) was measured after adding 5 mM ADP. PC1: Submaximal state 3 respiratory capacity specific to complex I was induced following the additions of 5 mM pyruvate and 10 mM glutamate. P: Maximal state 3 respiration, oxidative phosphorylation capacity was measured after the addition of 10 mM succinate. ETS: To measure the maximal electron transport system capacity by decoupling ATP synthase, we repetitively added 0.5 µM Carbonyl cyanide 4-(trifluoromethoxy)-phenylhydrazone until maximal rates of oxygen consumption was achieved. PC2: To measure the electron flow specific to complex II, we added 0.5 µM rotenone to inhibit complex I. We then added 2.5 µM antimycin A to inhibit complex III and to determine the residual, non-mitochondrial oxygen consumption, which was used for correcting the aforementioned measurements. COX: 2 mM ascorbate and 0.5 mM TMPD were simultaneously added to assess cytochrome c oxidase (COX) complex IV activity, which correlates with mitochondrial volume density (4) and was used to transform mass-specific respiration into mitochondria-specific respiration.

*Isolation of protein and mRNA from FFPE tissue sections*

FFPE tissue sections cut directly from blocks were deparaffinized and protein was isolated using Qproteome FFPE tissue kit (Qiagen #37623), according to manufacturer's instructions, followed by protein quantification and WB. For nucleic acid isolation, FFPE tissue sections directly cut from blocks were firstly deparaffinized by deparaffinization solution (Qiagen #19093) according to manufacturer's instructions, followed by total RNA and DNA purification using GenElute FFPE RNA/DNA Purification Plus Kit (Sigma Aldrich #RDP200) according to manufacturer's instructions.

# 2 Supplementary Figures and Tables

## 2.1 Supplementary Figures

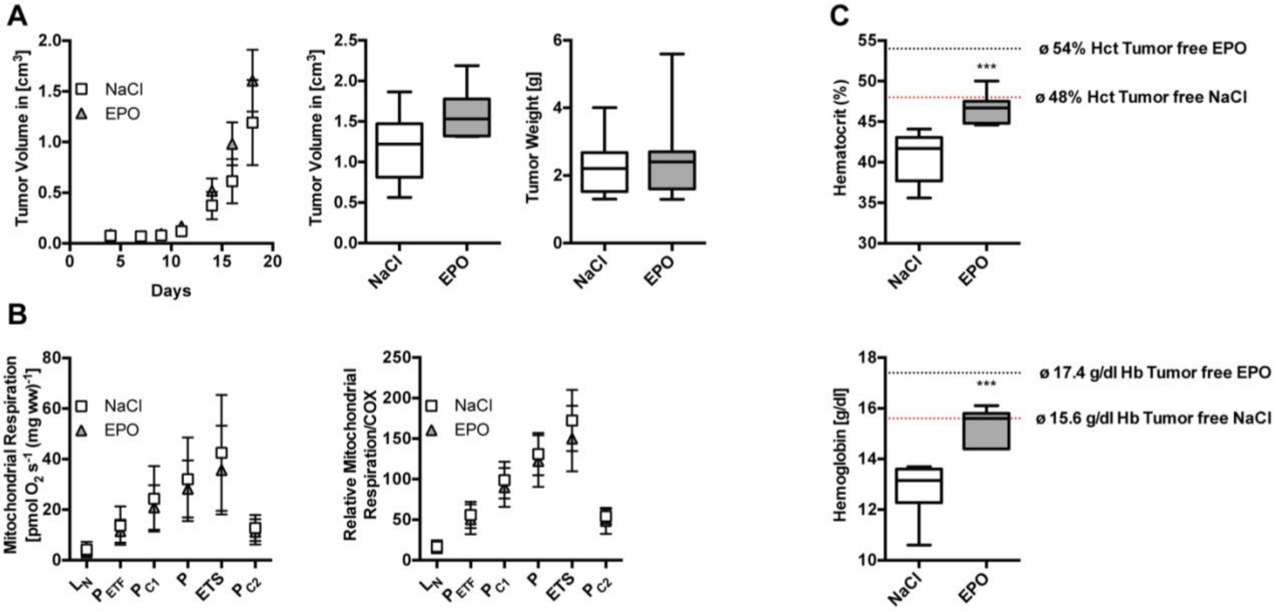


**Supplemental Figure 1. Erythropoietin treatment does not affect tumor growth and mitochondrial respiration in A549 lung cancer xenografts in Foxn1nu mice.** A549 lung cancer cells were subcutaneously injected (3 x 106 cells in 100 µl PBS/matrigel) into 16 Foxn1nu mice. Seven days after tumor implantation, mice were intraperitoneal (i.p.) injected with either 0.9% NaCl solution (white symbols) or 300 U/kg erythropoietin (EPO) (grey symbols) three times a week until the end of the experiment (8 mice per group). (A) Shown is the tumor growth curve, postmortem tumor size, and postmortem tumor weight of A549 xenografts from mice treated with 0.9% NaCl (white symbols) or 300 U/kg EPO (grey symbols) (n=8). Panel (B) shows mass-specific mitochondrial respiration per unit weight of tumor tissue (left panel) and mitochondrial-specific respiration normalized to cytochrome c oxidase activity (COX) (right panel) of A549 tumors from 0.9% NaCl (white squares) or 300 U/kg EPO (grey triangles) treated mice (n=7). LN, respiration in absence of adenylates; PETF, capacity for fatty acid β-oxidation; PC1, submaximal state 3 respiration through complex I; P, maximal state 3 respiration - oxidative phosphorylation capacity; ETS, electron transport system capacity; PC2, submaximal state 3 respiration through complex II. (C) Shown is hematocrit (Hct) (upper panel) and hemoglobin (Hb) (lower panel) of mice treated with 0.9% NaCl (white boxes) or 300 U/kg erythropoietin (grey boxes) (n=6-8). The dotted lines show average Hct and Hb values of tumor-free

Foxn1nu mice either treated with 300 U/kg (upper, black line) or saline (lower, red line). Data are presented as means and standard deviations or as box plots with min to max whiskers and a Student's t-test was performed (***p<0.001).

Results: Our data show that EPO treatment did neither increase tumor growth (A) nor altered mitochondrial respiration (B). However, EPO increased both, hematocrit (Hct) and hemoglobin (Hb) in tumor-free mice and in mice with A549 lung cancer xenografts confirming the activity of EPO, which was used for the experiments (C). Saline-treated mice had lower Hct and Hb values than tumor-free mice (40.6 vs 48% and 12.8 vs. 15.6 g/dl) indicating that A549 xenografts induce cancer-associated anemia in Foxn1nu mice. Treatment with EPO increased the average hematocrit from 40.6 to 46.5% (p<0.001) and the average hemoglobin from 12.8 to 16.1 g/dl (p<0.001). However, EPO-treated mice with A549 tumors did not reach the Hct and Hb levels of tumor-free mice (48% vs. 54% and 15.6 g/dl vs. 17.4 g/dl).

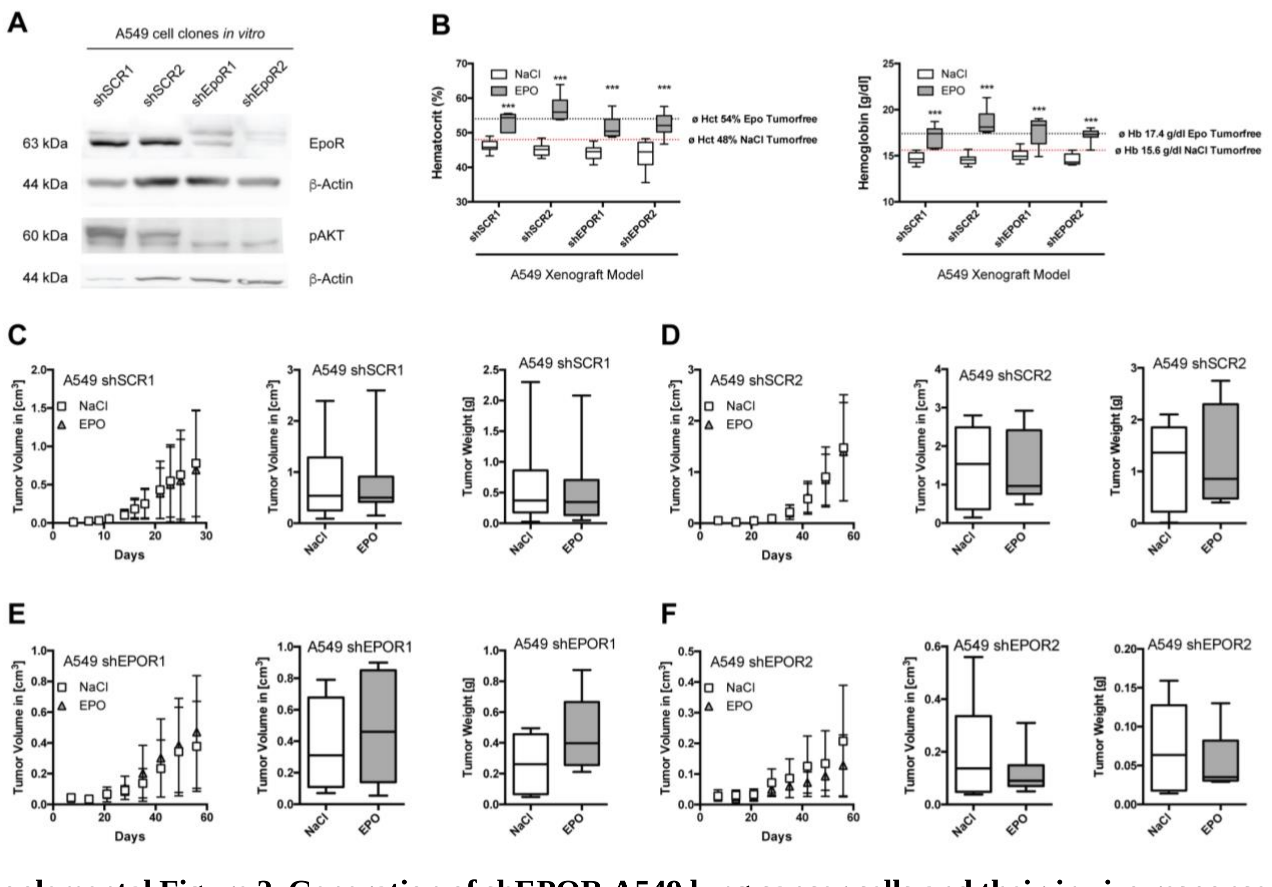


**Supplemental Figure 2. Generation of shEPOR A549 lung cancer cells and their *in vivo* response to EPO in Foxn1nu mice.** (A) We generated EPOR-knockdown A549 lung cancer cells by stable shRNA expression. A549 cells were infected with lentivirus to express either shSCR (control shRNA) or shEPOR (shRNA targeting EPOR). Because shEPOR expressing cells grew slower in vitro, shEPOR knockdown cells with a low knockdown efficiency (i.e., higher EPOR expression levels) were positively selected and outgrew cells with low EPOR expression levels (data not shown). Thus, we selected 2 individual clones from shSCR and 2 individual clones from shEPOR A549 cells and expanded them in vitro. Shown is a representative western blot image of EPOR (63 kDa), pAKT (60 kDa), and β-actin (44 kDa) protein expression of in vitro cultivated A549 shSCR1 and 2 and shEPOR1 and 2 knockdown clones. (B-F) A549 control cells (shSCR1 and shSCR2) or A549 EPOR-knockdown

cells (shEPOR1 and shEPOR2) were subcutaneously injected (3 x 106 cells in 100 µl PBS/matrigel) into immunocompromised Foxn1nu mice. 7 days after tumor implantation, mice were intraperitoneal (i.p.) injected 3 times a week with either 0.9% NaCl solution or 300 U/kg erythropoietin (EPO) until the end of the experiment. Panel (B) shows hematocrit (Hct) and hemoglobin (Hb) of 0.9% NaCl (white boxes) or 300 U/kg EPO (grey boxes) treated Foxn1nu mice, which were subcutaneously injected either with shSCR or shEPOR A549 cells. Data are shown as box plots with min to max whiskers and the dotted lines show average hematocrit and hemoglobin values of tumor-free Foxn1nu mice treated either with 300 U/kg (upper, black line) or saline (lower, red line) (n=6-8). A student`s t-test was used for data analyses. ***p<0.001. (C-F) Shown are tumor growth curves (left panel), postmortem tumor sizes (middle panel) as well as tumor weights (right panel) of (C) A549 shSCR1 xenografts, (D) A549 shSCR2, (E) A549 shEPOR1 and (F) A549 shEPOR2 xenografts from mice treated with either 0.9% NaCl (white squares) or 300 U/kg EPO (grey triangles) (n=6-8). Data are shown as box plots with min to max whiskers or as means and standard deviations (n=8). A Student’s t-test was used for data analyses.

Results: A549 EPOR knockdown cells showed lower EPOR protein levels than shSCR clones (A). EPO induced the mean Hct and mean Hb in mice, regardless of whether they carried shEPOR or shSCR A549 tumors (Hct: shSCR1 46.2 to 53.3%, shSCR2 45.1 to 57.0%, shEPOR1 44.3 to 51.6% and shEPOR2 43.7 to 52.2% (p<0.001); Hb: shSCR1 14.7 to 17.0 g/dl, shSCR2 14.6 to 18.7 g/dl, shEPOR1 15.1 to 17.7 g/dl and shEPOR2 14.6 to 17.2 g/dl (p<0.001)) (B). Although EPO increased hematopoiesis, it did not induce tumor proliferation in shEPOR or shSCR tumors (C-F).

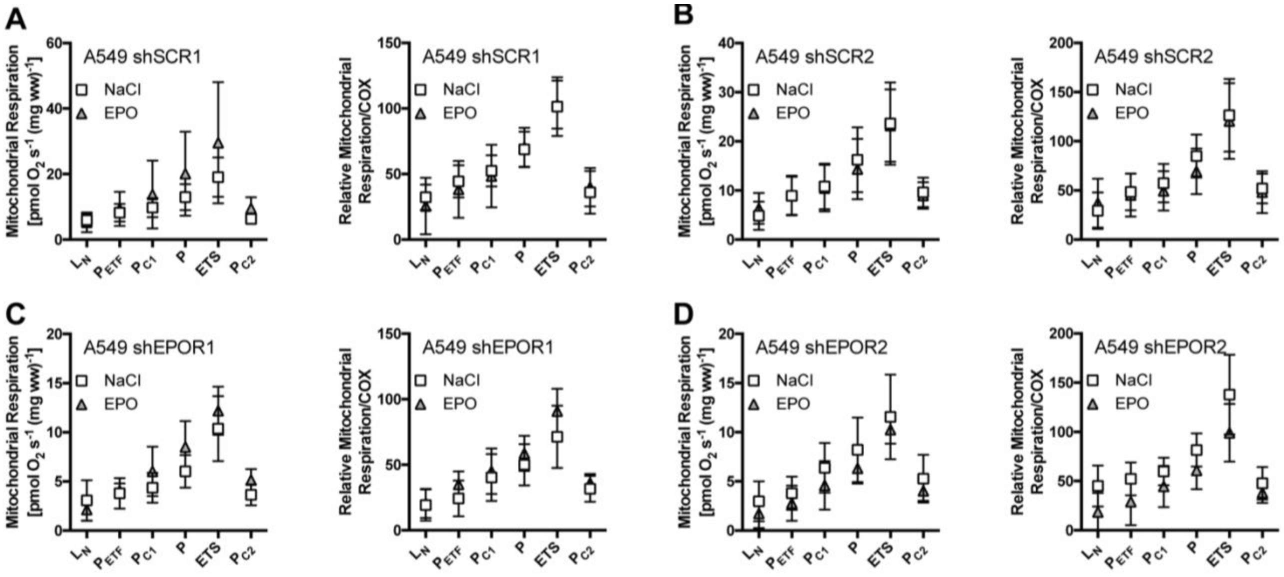


**Supplemental Figure 3. EPO does not impact mitochondrial respiration in shSCR and shEPOR A549 lung cancer xenografts in Foxn1nu mice.** A549 control cells (shSCR1 and shSCR2) or A549 EPOR knockdown cells (shEPOR1 and shEPOR2) were subcutaneously injected (3 x 106 cells in 100 µl PBS/matrigel) into immunocompromised Foxn1nu mice. 7 days after tumor implantation, mice were intraperitoneal (i.p.) injected 3 times a week with either 0.9% NaCl solution (white symbols) or 300 U/kg erythropoietin (EPO) (grey symbols) until the end of the experiment. Fresh biopsies of full-size tumors were isolated to measure mass-specific (per unit weight of tumor tissue) and mitochondria-specific mitochondrial respiration (normalized to cytochrome c oxidase (COX) activity) by high-

resolution respirometry. Panel (A) shows mass-specific (left panel) and mitochondria-specific (right panel) mitochondrial respiration of either 0.9% NaCl (white squares) or EPO (grey triangles) treated mice with A549 shSCR1 xenografts. Panel (B,C and D) show mass-specific and mitochondria-specific mitochondrial respiration of (B) A549 shSCR2, (C) shEPOR1 and (D) shEPOR2 xenografts from mice treated with either 0.9% NaCl (white squares) or EPO (grey triangles) (n=6-7). LN, respiration in absence of adenylates; PETF, capacity for fatty acid β-oxidation; PC1, submaximal state 3 respiration through complex I; P, maximal state 3 respiration - oxidative phosphorylation capacity; ETS, electron transport system capacity; PC2, submaximal state 3 respiration through complex II. Data are shown as mean and standard deviation (n=6-7) and a Student's t-test was used for data analyses.

Results: Our data show that EPO did neither impact mass-specific nor mitochondria-specific mitochondrial respiration in A549 shSCR and shEPOR tumor xenografts.

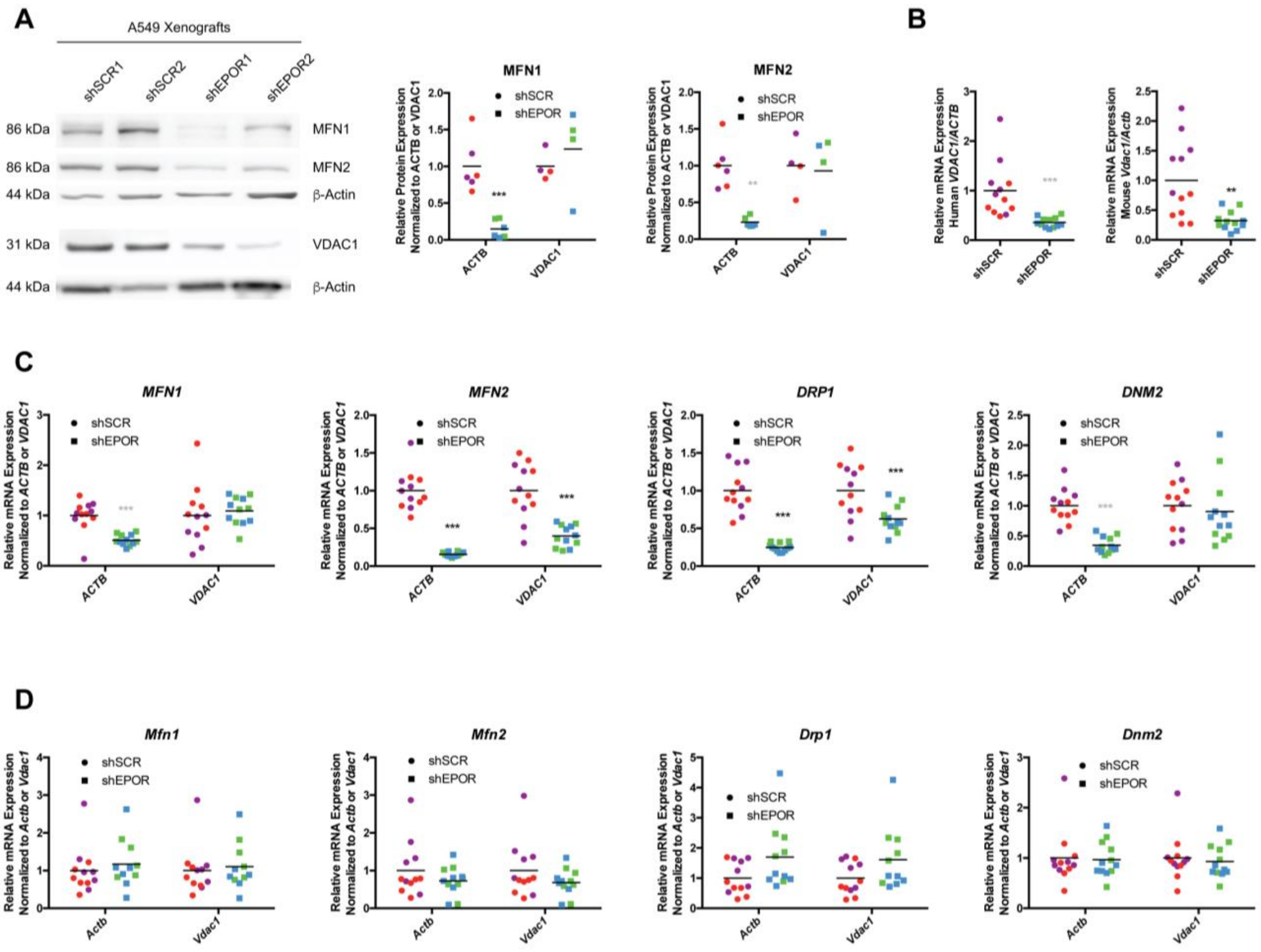


**Supplemental Figure 4. Effect of EPOR knockdown on mitochondrial dynamics in shSCR and shEPOR A549 lung cancer xenografts in Foxn1nu mice.** We analyzed expression levels of the main proteins and genes, which regulate mitochondrial dynamics, in control (shSCR) and EPOR knockdown (shEPOR) A549 tumor biopsies. Panel (A) shows a representative western blot image of mitofusin 1 and 2 (MFN1/2) 86 kDa, the main regulators of mitochondrial fusion, from protein extracts of control shSCR and shEPOR tumors. VDAC1 31 kDa (voltage-dependent anion-selective channel 1) was used as a marker for mitochondrial content and β-actin 44 kDa was used as a loading control. Protein levels of MFN1 and MFN2 from shSCR (purple clone 1, red clone 2) and shEPOR tumors (green clone 1,

blue clone 2) were quantified by measuring the band intensity of western blots with MCID Analysis 7.0. Expression was either normalized to β-actin, to estimate expression levels per cell, or to VDAC1 to normalize the MFN1 and 2 protein levels to mitochondrial content. (B) Human (left panel) and murine (right panel) mRNA levels of VDAC1 in shEPOR and shSCR tumors were quantified by qPCR and normalized to human or murine β-actin (ACTB). (C and D) Human (C) or murine (D) mRNA levels of MFN1 and MFN2 as well as the two mitochondrial fission genes DRP1 (DNM1L, dynamin-1-like protein) and DNM2 (dynamin 2) in shEPOR and shSCR tumors were analyzed by qPCR and normalized to either ACTB or VDAC1 mRNA levels. Data are shown as scattered blots with means (n=6-12) and individual data distribution of each clone (shSCR1 purple, shSCR2 red, shEPOR1 green, and shEPOR2 blue tumor samples) (n=6-12). Data were analyzed by a Student's t-test (black stars) or a Mann-Whitney test (grey stars). ***p<0.001; **p<0.01.

Results: MFN1 and MFN2 protein levels were 6.8 and 4.3 times lower in shEPOR tumors than in shSCR tumors when normalized to β-actin. However, when expression levels were normalized to VDAC1 no difference between shEPOR and shSCR tumors was observed. Thus, our data suggest that mitochondrial fusion was not or only little affected by EPOR knockdown in A549 tumors, because the ratio of mitofusin protein levels to mitochondria content in shEPOR tumors was similar to the ratio in shSCR tumors (A). Both, human and murine VDAC1 mRNA levels were down-regulated 2.8-fold (p<0.001) and 3.1-fold (p<0.01), respectively in shEPOR tumors (B). Thus, we normalized the mRNA expression of fusion and fission genes to ACTB (β-actin), to estimate the mRNA expression of these genes, as well as to VDAC1 to approximate the mRNA expression in relation to mitochondrial content. The data show that MFN1 normalized to ACTB was 2 times lower expressed in shEPOR tumors than in shSCR tumors (p<0.001) (C). In contrast, shEPOR and shSCR tumors did not differ in MFN1 expression when normalized to VDAC1. MFN2 in shEPOR tumors, however, was 6.4 times (p<0.001) and 2.5 times (p<0.001) lower expressed than in shSCR tumors when normalized to ACTB or VDAC1, respectively. Similarly, DRP1 expression in shEPOR tumors was 4.1 times (p<0.001) and 1.6 times lower (p<0.001) expressed in shEPOR than in shSCR tumors when normalized to ACTB or VDAC1, respectively. DNM2 expression in shEPOR tumors was 2.9 times lower (p<0.001) than in shSCR tumors but shEPOR and shSCR tumors did not differ in MNF1 expression when normalized to VDAC1 (C). The mRNA levels of murine Mfn1, Mfn2, Drp1, and Dnm2 did not differ between shEPOR and shSCR tumors, neither when normalized to Actb nor when normalized to Vdac1 (D). Thus, our data suggest, that the loss of EPOR in A549 lung cancer xenografts has little effect on mitochondrial dynamics: EPOR knockdown cells have lower MFN1/2 protein as well as MFN1/2, DRP1, and DNM2 mRNA expression levels than shSCR per cell (normalized to ACTB), however in relation to mitochondrial content (normalized to VDAC1) the ratio between mitochondrial content and expression of fusion and fission proteins or genes differs little between A549 shSCR and shEPOR tumors. Murine stromal cells did not show any difference between shSCR and shEPOR tumors. Thus, the regulation of mitochondrial dynamics seems to be independent of EPOR expression.

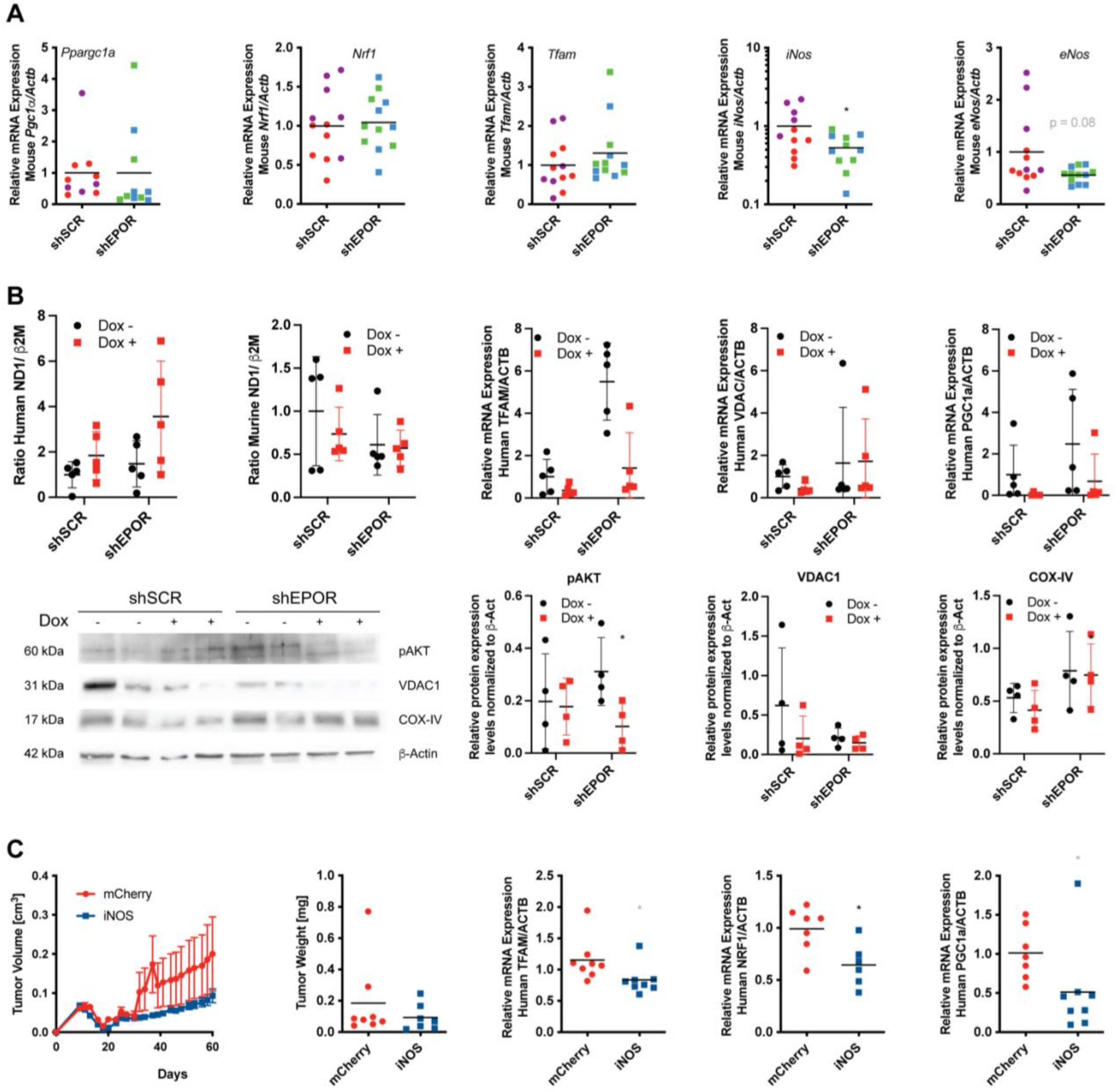


**Supplemental Figure 5. The effect of iNOS expression on mitochondrial biogenesis and nitric oxide synthase genes in A549 and MDA-MB-231 xenografts.** (A) We isolated biopsies of A549 tumors (including human cancer and murine stromal cells) grown in Foxn1nu mice either from human shSCR (control shRNA; purple, clone shSCR1; red, clone shSCR2) or from shEPOR (green, clone shEPOR1; blue, clone shEPOR2) EPOR-knockdown A549 cells. mRNA expression of the murine (i.e., gene expression in murine stromal cells only) mitochondrial biogenesis genes murine peroxisome proliferative activated receptor, gamma, coactivator 1 alpha (Pargc1a), nuclear respiratory factor 1 (Nrf1), and (transcription factor A, mitochondrial (Tfam), as well as iNos and eNOS, was analyzed using qPCR and normalized to β-Actin (Actb) (n=10-12). (B) Paraffin-embedded tumor tissues from a previous study on EPOR in MDA-MB-231 breast cancer tumors (5) were used to extract mRNA, gDNA, and proteins. In this study, mice were injected with tumor cells that expressed doxycycline-inducible (Dox) shSCR and shEPOR RNAs. Mitochondria content was determined by the ratio of human (1st panel) or murine (2nd panel) MT-ND1 (mitochondrially encoded NADH dehydrogenase

1) mitochondrial DNA to β2M (β-2microglobulin) genomic DNA in mice with shSCR or shEPOR tumors that were treated with doxycycline (red symbols) or not (black symbols). Further shown are mRNA expression levels of TFAM, voltage-dependent anion-selective channel 1 (VDAC1), and PGC1a analyzed by qPCR and normalized to ACTB. Further shown is a representative western blot image of proteins isolated from paraffin-embedded shSCR or shEPOR tumors. Antibodies against pAKT (60 kDa), VDAC1 (31 kDa), and COX-IV (17 kDa) as well as against the loading control marker β-actin (44 kDa) were used. Band intensities of proteins in western blot images were quantified using MCID Analysis 7.0 and normalized to β-actin. The relative protein expression levels of pAKT, VDAC1, and COX-IV are shown for control (shSCR) and EPOR knockdown (shEPOR) tumors that were treated with doxycycline (Dox) (red) or not (black) (n=4). (C) iNOS expression was rescued in A549 shEPOR1 knockdown cells by lentiviral delivery of mCherry (control) or iNOS to determine the impact of iNOS on tumor growth and mitochondrial biogenesis. A549 shEPOR1 control cells (mCherry, red symbols) or A549 shEPOR1 cells expressing iNOS (blue symbols) were subcutaneously injected (3 x 106 cells in 100 µl PBS/matrigel) into Foxn1nu mice. Shown is tumor growth as well as postmortem tumor weight. Also shown are mRNA expression levels of TFAM, NRF1, and PGC1a determined by qPCR and normalized to ACTB (n=7-8). Data are shown as scattered blots with means and individual data distribution or as means and standard deviations. A Student's t-test (black stars) or a Mann-Whitney test (grey p-value), as well as a 2-Way ANOVA, was performed. *$p<0.05$.

Results: Our data show that genes of mitochondrial biogenesis and nitric oxide synthesis were not differentially regulated in murine stromal cells of shEPOR A549 tumors. Only iNos mRNA levels in murine stromal cells of shEPOR A549 tumors were 1.9 times lower than in murine stromal cells of shSCR tumors ($p<0.05$) (A). This, however, is much less than in the human cancer cells of shEPOR tumors, where iNOS mRNA levels were 100 times lower than in shSCR A549 tumors (Fig.4), suggesting that mitochondrial biogenesis and NO synthesis are mainly impaired in human cancer cells of A549 tumors and less in murine stromal cells, although the mitochondrial content was reduced in both, human cancer, and murine stromal cells of shEPOR tumors (Fig. 4). Further, our data show that knocking down EPOR in iNOS deficient MDA-MB-231 tumors does not impact on mitochondrial content nor genes of mitochondrial biogenesis (B). This suggests that iNOS expression is required to mediate the effect of EPOR on mitochondrial biogenesis. However, the re-expression of iNOS in A549 EPOR knockdown cells does not stimulate mitochondrial biogenesis or tumor growth (C), suggesting that iNOS might be required but may act in concert with other factors (e.g., pAKT) to mediate the effect of EPOR on mitochondrial biogenesis.

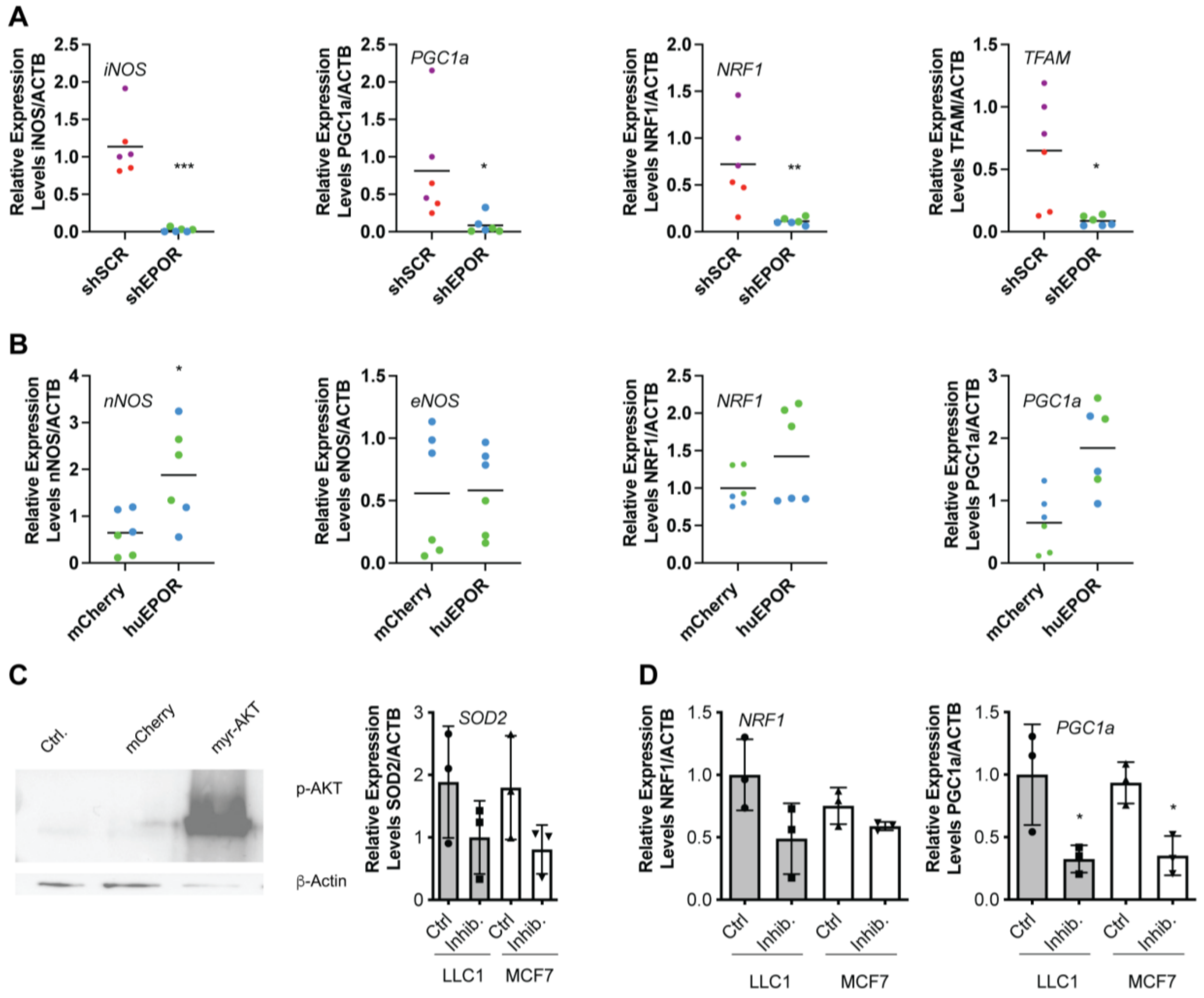


**Supplemental Figure 6. Supplementary *in vitro* data for EPOR rescue, pAKT overexpression, and iNOS/pAKT inhibition.** (A) Shown are mRNA levels of nitric oxide synthase 2 (iNOS), peroxisome proliferative activated receptor, gamma, coactivator 1 alpha (PGC-1a), nuclear respiratory factor 1 (NRF1), and transcription factor A, mitochondrial (Tfam) in in vitro cultured A549 shSCR (purple, clone shSCR1; red, clone shSCR2) and A549 shEPOR (green, clone shEPOR1; blue, clone shEPOR2) cells quantified by qPCR and normalized to β-actin (ACTB) mRNA. (B) We rescued EPOR expression in A549 shEPOR (green, clone shEPOR1; blue, clone shEPOR2) cancer cells by transfecting them with a human EPOR (huEPOR) overexpression plasmid in vitro for 72 h. Shown are mRNA levels of nitric oxide synthase genes nNOS and eNOS as well as NRF1 and PGC-1a from mCherry (control) and huEPOR transfected cells using qPCR and normalized to ACTB mRNA. (C) Shown is a representative western blotting image of A549 shEPOR1 lung cancer cells that were transfected with myr-AKT (Addgene plasmid #9005) or a mCherry expressing vector. β-Actin was used as a loading control. (D) Murine LLC1 lung cancer cells (grey bars) as human MCF-7 breast cancer cells (white bars) were either treated in vitro for 72 h with 200 µM L-NAME + 5 µM API-1 (Inhib.) to simultaneously inhibit iNOS and AKT or not (Ctrl.). Shown are RNA levels of superoxide dismutase 2 (SOD2), NRF1, and PGC1a quantified using qPCR and normalized to ACTB. Data are

shown as scattered blots with means and individual data with or without bars. A Student’s t-test (black stars) was performed (*p<0.05).

## 2.2 Supplementary Tables

**Supplemental Table 1 Antibodies**

| **Primary Antibody** | **Dilution** | **Supplier** |
|---|---|---|
| rat anti-human EPOR monoclonal antibody | 1:200 | Genovac, #GM-1201 |
| mouse anti-total OxPhos Complex monoclonal antibody | 1:250 | ThermoFisher Scientific, #458099 |
| rabbit anti-COX IV monoclonal antibody (3E11) | 1:1000 | Cell Signaling, #4850 |
| rabbit anti-AKT polyclonal antibody | 1:1000 | Cell Signaling, #9272 |
| rabbit anti-Phospho-AKT (Ser473) polyclonal antibody | 1:1000 | Cell Signaling, #9271 |
| mouse anti-iNOS (2D2-B2) monoclonal antibody | 1µg/ml | R&D, #MAB9502 |
| mouse anti-MFN1 (D-10) monoclonal antibody | 1:200 | Santa Cruz, #sc-166644 |
| mouse anti-MFN2 (XX-1) monoclonal antibody | 1:200 | Santa Cruz, #sc-100560 |
| rabbit anti-VDAC1 / Porin antibody – Mitochondrial Loading Control | 1:1000 | Abcam, #ab15895 |
| mouse anti-beta-actin monoclonal antibody | 1:5000 | Sigma Aldrich, #A5441 |
| **Secondary Antibody** | | |
| goat anti-rat | 1:5000 | Santa Cruz, #sc-2032 |
| donkey-anti-rabbit | 1:5000 | NA934V; Amersham |
| goat-anti-mouse | 1:5000 | Santa Cruz, #sc-2031 |

**Supplemental Table 2 Primer sequences**

| | |
|---|---|
| Human MT-ND1 | *F: 5'-GCTCTCACCATCGCTCTTCTACT-3'*<br>*R: 5'-ATTGTTTGGGCTACTGCTCGC-3'* |
| Human N-β2M | *F: 5'-CCAGCAGAGAATGGAAAGTCAA-3'*<br>*R: 5'-TCTCTCTCCATTCTTCAGTAAGTCAACT-3'* |
| Human *nNOS* | *F: 5'- CAGCGAGAAGGAGAAGCAGCG -3'*<br>*R: 5'- CTGGGTCAGGAGCAGGGTGG -3'* |
| Human *iNOS* | *F: 5'-ACGAGAAGCGGAGACCCAAG-3'*<br>*R: 5'-CATCAGCATACAGGCAAAGAGCA-3'* |
| Human *eNOS* | *F: 5'- GGAGGTGACAAGCCGCATAC -3'*<br>*R: 5'- GGAACTGGAAGGGAAAGCCA -3'* |
| Human *SOD1* | *F: 5'-TACAAAGACAGGAAACGCTGG-3'*<br>*R: 5'-CCTCAGACTACATCCAAGGGAA-3'* |
| Human *SOD2* | *F: 5'-AGG TGA CTC TAA CTT CCC TGG C-3'*<br>*R: 5'-CCCACAAGCACAGAAATAAAGGAGA-3'* |
| Human *SOD3* | *F: 5'-CGT TCC TGG GCT GGC TGG GT-3'*<br>*R: 5'-ATGGCTGGAGTCGGGCACCTTT-3'* |
| Human *CAT* | *F: 5'-GACATTACCAAATACTCCAAGGCAA-3'*<br>*R: 5'-AACCCGATTCTCCAGCAACA-3'* |
| Human *GPX3* | *F: 5'-AGGTATGCGTGATTGTGTGTGT-3'*<br>*R: 5'-GGAGAACTGGAGAGAAAGGGTTG-3'* |

| | |
|---|---|
| Human *GPX4* | *F: 5'-CGCTGTGGAAGTGGATGAAGA-3'*<br>*R: 5'-CTTGTCGATGAGGAACTTGGTGAA-3'* |
| Human *DNM1L* | *F: 5'-AGCAGTTTGCCTGTGGATAAG-3'*<br>*R: 5'-GCTTTCTTCTTGGAGGACTATGG-3'* |
| Human *DNM2* | *F: 5'-TTGGGCTATGTGGGTGGTGG-3'*<br>*R: 5'-AGTGGTCAAACTGAGCGAGGAG-3'* |
| Human *MFN1* | *F: 5'-CTTCTACTCCCACTGCTCCTACC-3'*<br>*R: 5'-AGATGTAACGGACGCCAATCCT-3'* |
| Human *MFN2* | *F: 5'-GTCCATCTTTCCACCCTCCCT-3'*<br>*R: 5'-CCCACTTCAACCAGTAACAATCCC-3'* |
| Human *PGC1a* | *F: 5'-CAAGCCAAACCAACAACTTTATCTCT-3'*<br>*R: 3'-CACACTTAAGGTGCGTTCAATAGTC-3'* |
| Human *TFAM* | *F: 5'-GAACAACTACCCATATTTAAAGCTCA-3'*<br>*R: 3'-GAATCAGGAAGTTCCCTCCA-3'* |
| Human *NRF1* | *F: 5'-GGCACTGTCTCACTTATCCAGGTT-3'*<br>*R: 5'-CAGCCACGGCAGAATAATTCA-3'* |
| Human *VDAC1* | *F: 5'-ACCAGAAAGTGAACAAGAAGTTGGAGA-3'*<br>*R: 5'-AAGCAGGCGTCAGGGTCAATC-3'* |
| Human *ACTB* | *F: 5'-CTGGAACGGTGAAGGTGACA-3'*<br>*R: 5'-AAGGGACTTCCTGTAACAACGA-3'* |
| Mouse MT-ND1 | *F: 5'-AGTCTATGAGTTCCCCTACCA-3'*<br>*R: 5'-GTGAGTATTTGGAGTTTGAGGC-3'* |
| Mouse N- β2M | *F: 5'-CGGCCTGTATGCTATCCAGA-3'*<br>*R: 5'-TCCACCCTGTAGCCTCAAAG-3'* |
| Mouse *nNos* | *F: 5'-GGTCAAGAACTGGGAGACAGA-3'*<br>*R: 5'-CTTCCGTATGTGATGGGATGG-3'* |
| Mouse *iNos* | *F: 5'-ACGAGACGGATAGGCAGAGATTGGA-3'*<br>*R: 5'-GACAGCAGGAAGGCAGCGGG-3'* |
| Mouse *eNos* | *F: 5'-AGG CTC TCA CCT ACT TCC TG-3'*<br>*R: 5'-TGAACCACTTCCATTCTTCGT-3'* |
| Mouse *Sod1* | *F: 5'-GCCTGTGGAGTGATTGGGATTG-3'*<br>*R: 5'-TAATGGTTTGAGGGTAGCAGATGAG-3'* |
| Mouse *Sod2* | *F: 5'-TCAAGATGGACAAAGTAGGTAGGG-3'*<br>*R: 5'-GCTGAATGGCTTCCCAGAATG-3'* |
| Mouse *Sod3* | *F: 5'-AACTTCACCAGAGGGAAAGAGC-3'*<br>*R: 5'-CAGTAGCAAGCCGTAGAACAAGAA-3'* |
| Mouse *Cat* | *F: 5'-GTCCGTCCCTGCTGTCTCAC-3'*<br>*R: 5'-CTGCTCCTTCCACTGCTTCATCT-3'* |
| Mouse *Gpx3* | *F: 5'-GTCCATCTGTGTTTACGGCTTG-3'*<br>*R: 5'-TAGAACTTGTGTTTCCTTTGGGCT-3'* |
| Mouse *Gpx4* | *F: 5'-TATGCTGAGTGTGGTTTACGAAT-3'*<br>*R:5'-TTGATTTCTTGATTACTTCCTGGCT-3'* |
| Mouse *Dnm1* | *F: 5'-AGGCATTACAAGGAGCCAGTCAAA-3'*<br>*R: 5'-GCAGCAGGTTCAAGTCA CAAAG-3'* |
| Mouse *Dnm2* | *F: 5'-AGAGAATGAGGATGGAGCACAAGAG-3'*<br>*R: 5'-TTTGGCATAAGGTCACGGATGGA-3'* |
| Mouse *Mfn1* | *F: 5'-AGCAAGGATGTAACCACTACTAAACA-3'*<br>*R: 5'-CTAAAGAGAGAGAGCAGGAAAGCA-3'* |

| | |
|---|---|
| Mouse *Mfn2* | *F: 5'-TAAAGGATGTAGCAGGAGGAATGG-3'*<br>*R: 5'-TGTAGCAAGGCAGGGATGAG-3'* |
| Mouse *Pgc1a* | *F: 5'-TGGGTGTGCGTGTGTGTATGT-3'*<br>*R: 5'-GTCGCCCTTGTTCGTTCTGTTC-3'* |
| Mouse *Tfam* | *F: 5'-AGCAGGCACTACAGCGATACA-3'*<br>*R: 5'-TCTACCTTTCCCATTCCCTTCCC-3'* |
| Mouse *Nrf1* | *F: 5'-AGCCTCCATCTTCTCCCTTCC-3'*<br>*R: 5'-AACACTTCTGTCACCTTCAGTTTGT-3'* |
| Mouse *Vdac1* | *F: 5'-GCTCACCTTTGATTCGTCATTCTC-3'*<br>*R: 5'-GGTTGATGTGCTCCCTCTTGT-3'* |
| Mouse *Actb* | *F: 5'-TTT CCA GCC TTC CTT CTT GGG-3'*<br>*R: 5'-GAGGTCTTTACGGATGTCAACG-3'* |

## 3 References

1. Faustino-Rocha A, Oliveira PA, Pinho-Oliveira J, Teixeira-Guedes C, Soares-Maia R, da Costa RG, et al. Estimation of rat mammary tumor volume using caliper and ultrasonography measurements. Lab Anim (NY). 2013;42(6):217-24.
2. Jacobs RA, Diaz V, Soldini L, Haider T, Thomassen M, Nordsborg NB, et al. Fast-twitch glycolytic skeletal muscle is predisposed to age-induced impairments in mitochondrial function. J Gerontol A Biol Sci Med Sci. 2013;68(9):1010-22.
3. Jacobs RA, Fluck D, Bonne TC, Burgi S, Christensen PM, Toigo M, et al. Improvements in exercise performance with high-intensity interval training coincide with an increase in skeletal muscle mitochondrial content and function. J Appl Physiol (1985). 2013;115(6):785-93.
4. Larsen S, Nielsen J, Hansen CN, Nielsen LB, Wibrand F, Stride N, et al. Biomarkers of mitochondrial content in skeletal muscle of healthy young human subjects. J Physiol. 2012;590(14):3349-60.
5. Chan KK, Matchett KB, Coulter JA, Yuen HF, McCrudden CM, Zhang SD, et al. Erythropoietin drives breast cancer progression by activation of its receptor EPOR. Oncotarget. 2017;8(24):38251-63.
